# Do Lifestyle Interventions Mitigate the Oxidative Damage and Inflammation Induced by Obesity in the Testis?

**DOI:** 10.3390/antiox14020150

**Published:** 2025-01-27

**Authors:** Ruben J. Moreira, Pedro F. Oliveira, Maria Angélica Spadella, Rita Ferreira, Marco G. Alves

**Affiliations:** 1 Institute of Biomedicine, Department of Medical Sciences (iBiMED), University of Aveiro, 3810-193 Aveiro, Portugal; rubenjesusmoreira@ua.pt; 2 LAQV-REQUIMTE, Department of Chemistry, University of Aveiro, 3810-193 Aveiro, Portugal; p.foliveira@ua.pt (P.F.O.); ritaferreira@ua.pt (R.F.); 3 Human Embryology Laboratory, Marília Medical School, Marília 17519-030, SP, Brazil; maspadella@gmail.com

**Keywords:** obesity, physical exercise, caloric restriction, sperm parameters, testosterone, oxidative stress, inflammation

## Abstract

Obesity results from a disproportionate accumulation of fat and has become a global health concern. The increase in adipose tissue is responsible for several systemic and testicular changes including hormone levels (leptin, adiponectin, testosterone, estrogen), inflammatory cytokines (increase in TNF-α and IL-6 and decrease in IL-10), and redox state (increase in reactive oxygen species and reduction in antioxidant enzymes). This results in poor sperm quality and compromised fertility in men with obesity. Lifestyle modifications, particularly diet transition to caloric restriction and physical exercise, are reported to reverse these negative effects. Nevertheless, precise mechanisms mediating these benefits, including how they modulate testicular oxidative stress, inflammation, and metabolism, remain to be fully elucidated. The main pathway described by which these lifestyle interventions reverse obesity-induced oxidative damage is the Nrf2-SIRT1 axis, which modulates the overexpression of antioxidant defenses. Of note, some of the detrimental effects of obesity on the testis are inherited by the descendants of individuals with obesity, and while caloric restriction reverses some of these effects, no significant work has been carried out regarding physical exercise. This review discusses the consequences of obesity-induced testicular oxidative stress on adult and pediatric populations, emphasizing the therapeutic potential of lifestyle to mitigate these detrimental effects.

## 1. Introduction

The European Association for the Study of Obesity (EASO) characterizes obesity as the disproportionate accumulation of fat, which can be detrimental to health [1]. An adult is considered to be obese when their body mass index (BMI) is equal to or greater than 30 kg/m^2^, whereas, in children and adolescents, their weight for height must be lower than 2 standard deviations below the WHO Child Growth Standards median [2]. In both age groups, the prevalence of obesity has increased more than four-fold between 1990 and 2022, accounting for 890 million and 160 million individuals with obesity, respectively [2].

Obesity triggers several deleterious effects on the human body, particularly regarding male reproductive health. These problems include dysfunction of the hypothalamic–pituitary–gonadal (HPG) axis and hypogonadism [3], disruption of spermatogenesis [4] and adverse alterations in sperm parameters [4,5,6,7,8]. As a consequence of overnutrition, the physiological metabolic processes are dysregulated, triggering a cluster of cardiovascular risk factors, such as hyperglycemia and insulin resistance, atherogenic dyslipidemia and hypertension, also known as metabolic syndrome markers [9].

Both obesity and metabolic syndrome are tightly related to the onset of systemic inflammation and increased oxidative stress, which also occurs in the testes being considered the main causes of male infertility [10,11,12,13]. Oxidative stress describes the imbalance between oxidant (reactive oxygen species—ROS) and antioxidant molecules towards the oxidant molecules, which react with cellular biomolecules, changing their structure and compromising their function [14]. Inflammation and molecules that induce oxidative stress associated with obesity are present in circulation and can reach the testis, disrupting the endocrine and paracrine signaling in Leydig and Sertoli cells. This disturbance compromises testosterone synthesis, spermatogenesis, and blood–testis barrier integrity, thereby impairing testicular function [15,16].

Regarding male reproductive physiology, the testis serves two critical functions: the production of spermatozoa and the synthesis of testosterone, both essential for male fertility and endocrine regulation [17,18]. These processes are tightly regulated by luteinizing hormone (LH) and follicle-stimulating hormone (FSH). LH acts on Leydig cells to stimulate testosterone synthesis [18], while FSH targets Sertoli cells, promoting their role in supporting spermatogenesis and maintaining the integrity of the blood–testis barrier [19]. The blood–testis barrier, formed by tight junctions between Sertoli cells, is a dynamic and highly specialized structure that separates the seminiferous tubule into basal and adluminal compartments. This barrier not only protects developing germ cells from immune surveillance by preventing the passage of systemic immune cells and antibodies but also creates a unique microenvironment necessary for spermatogenesis [20]. Sertoli cells also provide structural support, nutrients, and paracrine signals to germ cells and phagocytose residual cytoplasm during spermiogenesis [17]. Importantly, lactate dehydrogenase (LDH) activity, especially LDH-C, which is the main LDH isoform in Sertoli cells, supports testicular energy metabolism through glycolysis, producing lactate as a critical energy substrate for germ cell maturation [21].

In addition to inflammation and oxidative stress, other external factors, such as endocrine disruptors (EDs), are also significant contributors to testicular dysfunction. EDs, including obesogens [22], pesticides [23], and herbicides [24], can mimic or interfere with hormonal signaling, disrupting the physiological functions of the testis. Many of these compounds possess estrogen-mimetic properties, which can directly affect Leydig cells, reducing testosterone synthesis, and Sertoli cells, impairing spermatogenesis and compromising the integrity of the blood–testis barrier [25]. Furthermore, some EDs, such as obesogens, contribute to further increasing adiposity by promoting adipocyte differentiation and lipid storage, exacerbating the metabolic dysfunction associated with obesity [22]. Other EDs, due to their lipophilic nature, are stored in the expanding adipose tissue, which acts as a reservoir, prolonging their bioavailability and potential for endocrine disruption [26]. This is a topic widely studied, and although it is not the primary focus of our review, it represents a concerning issue when discussing the impacts of obesity.

Even though the effects of inflammation and oxidative stress are a concerning issue, some studies have shown that it is possible to reverse or mitigate these obesity-related complications through lifestyle alterations, specifically dietary corrections and regular physical activity [27,28].

With this literature review, we aim to discuss the current knowledge on the consequences of obesity-induced oxidative stress on testicular health, explore how this can be reversed or mitigated by lifestyle alterations, and the possible risks of these behavioral modifications. We summarize key findings, identify emerging trends, and highlight possible literature gaps.

## 2. How Does Obesity Fuel Systemic Oxidative Stress?

In this chapter, we summarize the current understanding of how obesity induces inflammation and oxidative stress, both systemically and within the testis. We discuss how these processes interact and contribute to alterations in systemic and testicular hormonal profiles. In later sections of this review, we will examine how these changes impair male fertility.

### 2.1. The Co-Dependence Between Inflammation and Oxidative Stress

Obesity is a metabolic disease that significantly impacts systemic homeostasis, leading to insulin resistance, hyperglycemia, and other metabolic disturbances such as dyslipidemia. These alterations stem from the excessive accumulation of adipose tissue, which disrupts endocrine and paracrine signaling, impairing glucose uptake and promoting chronic hyperinsulinemia and beta-cell dysfunction. Such metabolic derangements set the stage for the development of type 2 diabetes mellitus and other obesity-related complications [29,30].

Additionally, obesity is considered a metabolic disease characterized by low-grade inflammation [31]. As such, it displays several specific hallmarks of inflammation, due to (1) its metabolic nature, triggered by specialized cells (namely adipocytes); (2) its localized origin (when compared to infections or other acute immune responses); (3) the alteration of the cellular and molecular composition of tissues with substantial immune cell infiltration; and (4) lack of resolution of the inflammatory response, leading to chronicity [31]. Considering this chronic, localized, and low-grade inflammation, several pro-inflammatory cytokines are increased in individuals with obesity, including Tumor Necrosis Factor-alpha (TNF-α), Interleukin-6 (IL-6), Interleukin-7 (IL-7), Interleukin-8 (IL-8), Interleukin-1 (IL-1), monocyte chemoattractant protein-1 (MCP-1), and plasminogen activator inhibitor-1 (PAI-1). On the contrary, IL-10—an anti-inflammatory cytokine—is reduced in obesity [32]. Of note, inflammation and oxidative stress are related in such a way that when one appears as a primary disorder the other develops as a secondary disorder. This connection between inflammation and oxidative stress comes from the fact that immune cells that release inflammatory cytokines also tend to release ROS. Furthermore, cytokines such as TNF-α and IL-6 mediate the expression of ROS-producing enzymes, such as NADPH oxidase (NOX). On the other hand, oxidative stress tends to induce the activation of the NOD-like receptor protein 3 (NLRP3) inflammasome, which triggers leukocyte chemotaxis and inflammatory cytokine production [33]. Thus, there is clearly a co-dependence between these two processes in individuals with obesity.

### 2.2. Altered Adipokine Levels Cause Oxidative Stress in Individuals with Obesity

The nutrient and energy surplus cause the adipose tissue to grow, either by hyperplasia (adipocyte number increase) and/or hypertrophy (adipocyte size increase) [34,35]. These cellular alterations are concomitant with alterations in the secretion of adipokines—hormones from the adipose tissue. Leptin and adiponectin levels are particularly altered, and although these hormones can be produced in other organs (such as the skeletal muscle [36,37]), obesity has a significant effect on these hormone levels, given that they are primarily produced in the white adipose tissue [38]. Leptin is known as a satiety hormone, stimulating metabolic reactions that boost energy expenditure [39], whereas adiponectin is an insulin sensitizer [40,41] since it stimulates glucose catabolism and fatty-acid oxidation by activating AMP-activated protein kinase (AMPK) [42]. Adiponectin also regulates inflammation in a context-dependent manner since when there is an inflammatory state it promotes macrophage polarization into M2 phenotype but when an inflammatory response is needed, it induces pro-inflammatory cytokine production in M1 macrophages [43,44,45]. While leptin levels increase in obesity [46], adiponectin levels decrease abruptly [47], which at first glance may seem paradoxical. On one hand, since the cells that produce these hormones increase, so should the hormones, as occurs with leptin, but not with adiponectin. However, leptin stimulates the reduction in food intake and metabolic expenditure, so it should not be increased in a condition such as obesity. The answer to this conundrum relies on the fact that in a hyperleptinemic state, the maximum hypothalamic leptin response is decreased, resulting in leptin resistance [48], and adiponectin is reduced by processes inherent to obesity, which will be discussed during this review. Hence, a lower adiponectin/leptin ratio serves as a strong indicator of oxidative stress and inflammation associated with conditions that induce major metabolic dysfunction such as obesity and metabolic syndrome [49].

The excessive adiposity typical of obesity directly and indirectly hampers the production of adiponectin. The direct hampering comes from nutrient overload, which promotes excessive protein synthesis in several tissues, which leads to the accumulation of misfolded proteins in the adipocyte, especially adiponectin [50]. This prompts endoplasmic reticulum (ER) stress and activates the unfolded protein response (UPR)—a response that follows the PERK (protein kinase R-like ER kinase), IRE1 (inositol-requiring enzyme 1), and/or ATF6 (activating transcription factor 6) pathways [51]. Indirectly, the expansion of the adipose tissue reduces adiponectin production through hypoxia- and inflammatory-related mechanisms. Obesity-induced hypoxia [52] further boosts ER stress, mostly by triggering the IRE1 and PERK pathways of the UPR [53]. The IRE1 pathway includes the XBP1 effector, which is involved in folding, maturing, and degrading unfolded proteins [54]. Similarly, the PERK pathway includes the initiation Factor 2alpha (eIF2α) and the CCAAT-enhancer-binding protein homologous protein (CHOP) effectors [54]. In cases of hypoxia, the IRE1/XBP1 and the PERK/eIF2α/CHOP axes are mostly activated for the degradation of misfolded adiponectin [53]. The eIF2α effector also stimulates factor nuclear kappa B (NF-κB) to promote the expression of IL-6 in adipocytes. The expression of this cytokine inhibits the production of adiponectin [55]. Furthermore, CHOP also directly inhibits the transcription of adiponectin [56]. Another consequence of adipose hypoxia is the stabilization and activation of hypoxia-inducible factor 1*α* (HIF-1α) and subsequent expression of plasminogen activator inhibitor (PAI)-1 [57]. Additionally, the expanding adipose tissue directly produces MCP-1 [58], which not on contributes to the establishment of systemic insulin resistance by undermining insulin signaling at Akt phosphorylation and ERK activation but is also associated with increased adipose inflammation [59]. The IRE1 pathway, PAI-1 and MCP-1 work in tandem to promote infiltration and polarization of M1 macrophages in the white adipose tissue [60], increasing inflammation by synthesizing TNF-α and IL-6 [59,60,61]. Interestingly, PAI-1 is also expressed in rat Sertoli cells [62] and is positively regulated by testicular macrophage-produced TNF-α [63], indicating a positive feedback between these factors, which potentiates testicular inflammation. Thus, obesity reduces adiponectin levels by causing ER stress via the PERK and IRE1 pathways and by inducing inflammation dependently and independently of hypoxia.

One of the major ways hyperleptinemia and hypoadiponectinemia trigger systemic oxidative stress is by increasing the activity of the NOX. NOX is involved in the polyol pathway to catalyze the reduction in glucose to sorbitol with NADPH consumption. Considering that NADPH is utilized in the regeneration of reduced glutathione (GSH)—an important antioxidant—its depletion in high glucose conditions compromises the fine oxidant/antioxidant balance, tipping the scale in favor of the oxidant status [64]. In phagocytes, NOX2 is dedicated to the production of ROS, especially superoxide (O_2_^•−^) [65], and considering the previously described increase in phagocytes in the adipose tissue this might be a mechanism by which adipose macrophages induce oxidative stress in obesity. Increased NOX2 was also found in the testis of rats fed with a high-fat diet (HFD) [66]. Other isoforms are distributed across the body and respond to the changes induced by obesity. For instance, NOX4 in combination with IL-1β is associated with diabetic gastrointestinal complications and the onset of colorectal cancer [67]. Furthermore, NOX1 is responsible for the production of O_2_^•−^ in renal vascular and endothelial cells, inducing oxidative stress in the kidney [68]. Interestingly, in the kidney, NOX4 reduces hydrogen peroxide (H_2_O_2_), displaying a protective effect [68], which indicates a tissue-dependent effect of each NOX isoform. Leptin and adiponectin boast contrary effects regarding NOX expression and function. Whereas leptin tends to increase NOX isoforms overactivity and circulating O_2_^•−^ [69], adiponectin inhibits NOX expression and function across multiple tissues (especially NOX4) [70]. Taking this into account, it is easy to understand that obesity upholds mechanisms that favor NOX overexpression and overactivation, boosting systemic oxidative stress. A study by Furukawa and colleagues concluded that the overproduction of ROS in the expanding adipose tissue by NOX and the downregulation of antioxidant enzymes serve as a response to deter further lipid accumulation. This oxidative response, headed by NOX4, detrimentally dysregulates adipokine production and increases ROS circulation in peripheral blood, which ultimately culminates in insulin resistance in several tissues, hampered insulin secretion, and vascular diseases (atherosclerosis and hypertension), all characteristic of obesity [71].

In the testis, the expressed isoforms of NOX are NOX4 [72] and NOX5 [72,73]. Although their functions remain elusive, NOX5 is expressed in pachytene spermatocytes, round spermatids and spermatozoa, and has been associated with the putative generation of physiological levels ROS [73]. Therefore, it could be hypothesized that NOX5 may also be involved in excessive ROS production and testicular oxidative stress caused by obesity. Another enzyme involved in testicular oxidative stress and inflammation in obesity is the myeloperoxidase, a heme-containing peroxidase expressed mainly by neutrophils and monocytes that produce high levels of ROS, usually to kill microbial agents, however commonly linked to tissue damage [74]. This marker is increased in both serum and testicular tissue of Wistar albino rats fed with an HFD [75] and is concomitant with the expression of NOX2 in the testis [66]. Thus, the hormonal profile of obesity includes hyperleptinemia and hypoadiponectinemia. These alterations in leptin and adiponectin are linked to systemic and localized increases in oxidative stress, mediated mainly by NOX isoforms and myeloperoxidase, and inflammation, mediated by TNF-α and IL-6, among other cytokines.

### 2.3. Oxidative Phosphorylation, Fatty Acid Oxidation and Pentose Phosphate Pathway in Obesity-Induced Systemic Oxidative Stress

In the liver, several metabolic pathways are upregulated by obesity. These include gluconeogenesis, peroxisomal fatty acid oxidation, Krebs cycle, oxidative phosphorylation, and the pentose phosphate pathway [76].

In the scope of this review, the pentose phosphate pathway, the peroxisomal fatty acid oxidation, and the oxidative phosphorylation are the most noteworthy. The pentose phosphate pathway requires NADP^+^ for the conversion of glucose-6-phosphate to 6-phosphogluconolactone, catalyzed by the glucose 6-phosphate dehydrogenase. This reaction reduces NADP^+^ to NADPH, which could be used by NOX isoforms to produce O_2_^•−^ [77], as previously discussed.

Peroxisomes and mitochondria hold a tight interplay regarding fatty acid oxidation, with each one being responsible for oxidizing different sets of lipids [78]. Usually, peroxisomes are in charge of metabolizing very-long-chain fatty acids [79], with their final products being shuttled to the mitochondria, which in turn reoxidizes NADH to NAD^+^ for the peroxisomal reaction to continue [78]. Since peroxisomes lack a second membrane and the electron transport chain, electrons are transported by FADH_2_ and delivered to oxygen, thus generating H_2_O_2_ [80].

Several genes from oxidative phosphorylation are upregulated by obesity and type 2 diabetes mellitus. Buchner and colleagues showed that 56 of the 76 oxidative phosphorylation genes were upregulated in mice fed a high-fat simple carbohydrate diet for 100 days [81]. This upregulation was evident across all five oxidative phosphorylation complexes and resulted in an increased mitochondrial oxidative capacity in the liver of mice with obesity [81]. Additionally, Pospisilik and colleagues demonstrated that moderate oxidative phosphorylation deficiency may protect against obesity and diabetes. The authors proved this with apoptosis-inducing factor (AIF) knockout mice. Despite its name, AIF is primarily a regulator of oxidative phosphorylation, and when depleted, the weight gain is significantly lower than in HFD-fed mice, mostly due to reduced activity of complexes I, IV, and V, and consequently shifting to the less efficient anaerobic glucose metabolism and fatty acid oxidation, thus increasing energy fuel expenditure [82].

All of the described increases in oxidative metabolism and NADPH-dependent pathways that occur especially in the liver, in conjunction with increased NOX activity and decreased antioxidant enzymes (such as catalase—CAT or glutathione peroxidase—GPx), serve as a driving force for systemic oxidative stress and tissue damage [76], including testicular damage, as will be explored in greater detail in following chapters of this review.

**Figure 1:**
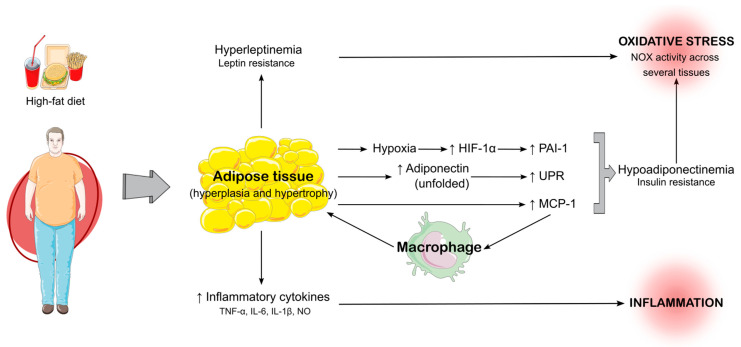
Mechanisms linking high-fat diet to adipose tissue expansion and systemic effects of inflammation and oxidative stress. A high-fat diet leads to hyperplasia and hypertrophy of adipose tissue. This expansion contributes to hyperleptinemia and leptin resistance. Enlarged adipose tissue experiences hypoxia, which activates hypoxia-inducible factor 1-alpha (HIF-1α) and increases plasminogen activator inhibitor-1 (PAI-1). Additionally, unfolded adiponectin triggers the unfolded protein response (UPR), especially the PERK (protein kinase R-like ER kinase) and IRE1 (inositol-requiring enzyme 1) pathways, responsible for the degradation of misfolded adiponectin and consequent hypoadiponectinemia and insulin resistance. Macrophages infiltrate the adipose tissue Adipose tissue also synthesizes monocyte chemoattractant protein-1 (MCP-1), which disturbs insulin signaling by disrupting Akt phosphorylation. MCP-1 contributes to inflammation by secreting inflammatory cytokines such as Tumor Necrosis Factor-alpha (TNF-α), Interleukin-6 (IL-6), Interleukin-1β (IL-1β), and nitric oxide (NO). Inflammatory and oxidative stress markers, including NADPH oxidase (NOX) activity, exacerbate systemic inflammation and oxidative stress. Together, these pathways contribute to dysfunctions associated with metabolic syndrome. ↑—upregulation.

Obesity is a complex metabolic condition that triggers both systemic inflammation and oxidative stress, which are intricately linked and mutually reinforcing. The disruption of normal adipokine signaling, particularly the imbalance between leptin and adiponectin, plays a central role in promoting oxidative stress and inflammation. These processes are compounded by the expansion of adipose tissue, which induces endoplasmic reticulum stress and further exacerbates systemic metabolic dysregulation. The chronic oxidative stress resulting from these interactions has consequences, not only for metabolic health but also for organs such as the testes, where it can impair reproductive function. A deeper understanding of the molecular mechanisms behind obesity-induced oxidative stress offers valuable insights into potential therapeutic strategies, including lifestyle interventions, to mitigate these harmful effects and restore metabolic balance. Figure 1 summarizes the mechanisms linking a high-fat diet to adipose tissue expansion and systemic effects, including inflammation and oxidative stress, as documented in the literature.

## 3. Oxidative Stress Impairs Spermatogenesis and Steroidogenesis

As observed in other tissues, obesity also induces inflammation in the testes, mainly through the overexpression of TNF-α, inducible nitric oxide synthase (iNOS), and IL-1β, accompanied by the underexpression of IL-10 [83]. This inflammation is associated with the inhibition of steroidogenesis in Leydig cells [84]. Among these inflammatory mediators, TNF-α is a particularly strong negative modulator of steroidogenesis. This modulation occurs via the Jun N-terminal kinase 1/extracellular regulated protein kinase/mitogen-activated protein kinase (JNK/ERK/MAPK) pathway [85] and the expression of NF-κB [86]. The JNK/ERK/MAPK pathway induces the expression of dosage-sensitive sex-reversal–adrenal hypoplasia congenita critical region on the X chromosome, gene 1 (DAX-1), which represses several transcription factors involved in steroidogenesis, in particular the orphan nuclear receptors nuclear receptor subfamily 4 group A member 1 (Nur77) and steroidogenic factor 1 (SF-1) [85,87,88]. NF-κB directly inhibits the transactivation of both Nur77 and SF-1 [86]. These receptors are essential for the expression of several enzymes involved in steroidogenesis following luteinizing hormone (LH) activation in Leydig cells [89,90], most notably steroidogenic acute regulatory protein (StAR) [89,91], which catalyzes the rate-limiting entrance of cholesterol into the mitochondria [92,93], but also cytochrome P450, family 11 (Cyp11a; only SF-1 [91]), Cyp17a (both [94,95]), and 3-beta-hydroxysteroid dehydrogenase (3β-HSD; both [96,97]). They carry out this activation by binding to the promoter region of each gene after LH-induced cyclic adenosine monophosphate (cAMP) stimulation [89]. SF-1 is also a key player in fetal Leydig cell differentiation and adult Leydig cell survival [98]. Therefore, the inhibition of these receptors severely disrupts steroidogenesis.

The disruption of testosterone production in obesity, metabolic syndrome and diabetes is well documented in the literature, with both serum [11,99,100] and intratesticular levels [99,100] being reduced in rats with obesity and serum levels of men with obesity [101,102]. In contrast, estrogens tend to be increased in men with obesity [101], with significant correlations found between the testosterone/estradiol ratio and body weight [103]. This ratio is an indicator of overall metabolic health in men, where higher levels are associated with higher high-density lipoprotein (HDL) and lower total cholesterol and circulating triglycerides, while lower levels correlate with poorer metabolic parameters [103]. This androgen-to-estrogen shift occurs primarily in the expanding white adipose tissue due to TNF-α-induced overexpression of aromatase (CYP19A1)—an enzyme that catalyzes the irreversible aromatization of testosterone into estradiol [104,105].

In physiological conditions, androgens and estrogens modulate LH secretion via negative feedback, ensuring controlled sex steroid production. However, estrogens are more potent suppressors of LH, so their elevated concentrations in obesity lead to stronger LH suppression. Consequently, the *Nr0b2* gene (which is suppressed by the active LH pathway via AMPK) is expressed and the NR0B2 protein accumulates, inhibiting transcription factors (NR5A1 and NR5A2) that regulate StAR expression [106]. This results in testosterone levels being negatively impacted by free estradiol levels [107]. Additionally, Leydig cells may also express aromatase when the testicular testosterone/estradiol ratio is decreased [108]. This phenotypical change creates a positive feedback loop, exacerbating the decline in testosterone. In HFD-fed mice (8 or 19 weeks), testosterone is not only lower than standard chow-fed mice, but this reduction is also associated with high oxidative stress, as evidenced by increased malondialdehyde (MDA), nitric oxide (NO), and H_2_O_2_ and decreased CAT and GPx (19 weeks only) [109]. In fact, a study by Polari and colleagues showed that a plant-based polyphenol mixture with antioxidative and anti-inflammatory properties reduced white adipose tissue aromatase expression [104]. Thus, therapeutic strategies targeting inflammation and oxidative stress may be helpful to improve testosterone levels in individuals with obesity.

Along with aromatase, estrogen sulfotransferase, which is expressed discretely in Leydig cells and the corpus and cauda of the epididymis, is responsible for the sulfonation and consequent inactivation of estrogens [110]. The activity of this enzyme allows for the control of excessive estrogens, such as estradiol, and mitigates estrogen-mediated reduction in sperm motility [110]. Estrogen sulfotransferase is associated with improved metabolic status in males, as well as reduced inflammation and increased insulin sensitivity [111]. It has been reported that it is also associated with the increase in antioxidant defenses, for instance in cases of breast cancer, mostly by upregulating Nrf2 (nuclear factor erythroid 2–related factor 2) [112]. This enzyme is also expressed in the liver and adipose tissue and is overexpressed during obesity [113] and the depletion of its gene exacerbates the diabetic phenotype in male mice [114].

In obesity, the testicular iNOS enzyme is overexpressed [83], resulting in an overproduction of NO [11]. Within the testis, NO inhibits the activity of CYP11A1, an essential endoplasmic reticulum enzyme involved in steroidogenesis [115], and decreases sperm progressive motility [116]. NO may react with O_2_^•−^ to form reactive nitrogen species (RNS) [116], such as peroxynitrite, which not only decreases sperm motility but also disrupts mitochondrial membrane potential [117], indicating that obesity causes nitrosative stress as well as oxidative stress.

Hyperleptinemia and hypoadiponectinemia also contribute to reduced testosterone levels. Leptin was found to be inversely correlated with testosterone in HFD-fed mice (8 or 19 weeks) [107,109] since it favors androgen-to-estrogen conversion in the adipose tissue [7]. Conversely, adiponectin and adiponectin receptor 1 (AdipoR1)—both present in adult mice testes and in particular in Leydig cells [118]—increase testosterone production, by directly stimulating the expression of steroidogenic enzymes and indirectly by enhancing expression of antioxidant enzymes CAT and GPx and decreasing lipid peroxidation [119]. AdipoR2 is mostly expressed in germ cells of the seminiferous tubules [118] and its genetic knockout results in seminiferous tubules atrophy and consequent reduction in testicular weight, although testosterone levels remained the same [120]. It was not clear whether knocking out AdipoR1 had an effect on testosterone synthesis; however, it favored an obese and insulin resistance phenotype [120].

As discussed, obesity fuels oxidative stress by increasing oxidant species; however, it also represses antioxidant defenses, particularly via the Keap1-Nrf2-ARE (Kelch-like ECH-associated protein 1–nuclear factor erythroid 2-related factor 2–antioxidant response element) signaling pathway. In physiological conditions, Nrf2, a transcription factor, is sequestered and inactivated by Keap1. During oxidative stress, the Keap1-Nrf2 complex disengages in a dose-dependent manner, allowing Nrf2 to translocate to the nucleus, where it heterodimerizes with small musculoaponeurotic fibrosarcoma oncogene homolog (Maf) proteins. This complex activates antioxidant response element (ARE)-regulated genes which encode several antioxidant and cytoprotective proteins, including heme oxygenase-1 (HO-1—reduced NF-κB inflammatory activity), NAD(P)H dehydrogenase (promotes synthesis and regeneration of NADPH, contradicting NOX activity), superoxide dismutase (SOD—catalyzes the dismutation of O_2_^•−^), NAD(P)H:quinone oxidoreductase (NQO—catalyzes the conversion of quinones to less toxic molecules), and CAT (reduces H_2_O_2_ to water and oxygen) [121]. In HFD-fed male rats (12 weeks), testicular Nrf2 mRNA and antioxidant enzyme expression (CAT, SOD, GPx) were significantly reduced. However, these results were mitigated after treatment with an antioxidant agent (orlistat) [83], proving that antioxidant therapies should be considered as a treatment for obesity. Similarly, in HFD-fed male mice (5 months), germ cells exhibited high NOX4 expression and reduced Nrf2-Keap1-HO1 axis activity, leading to germ cell apoptosis [122]. Moreover, in C57BL/6J male mice with HFD (19 weeks), Nrf2 mRNA expression in Leydig cells was also downregulated, along with CAT, SOD, and GPx expression and activity [109]. Thus, Nrf2 and its capacity to maintain the intracellular oxidant/antioxidant balance helps in safeguarding steroidogenesis (along with SIRT1, as will be explained further in this review) [123]. In humans, low seminal Nrf2 mRNA expression was correlated with low SOD expression and poor sperm motility [124]. It would be interesting to analyze the relationship between Nrf2 expression in semen and other sperm parameters, as Nrf2 seems to hold importance in the regular functioning of steroidogenesis and spermatogenesis.

Obesity and oxidative stress impact sperm cells in such a way that sperm parameters are altered. Animal and human studies have reported impairment of sperm count [4,102], concentration [4,5,102], motility [27] (especially progressive motility [5,102]), normal morphology [102] (decreased in detriment of abnormal morphology [27,125]), sperm viability [27], and semen volume [4]. Elevated ROS in semen is associated with poorer sperm quality [126]. Interestingly, ROS plays a dual role in sperm function. While controlled ROS levels are necessary for processes like capacitation, excessive ROS are detrimental to fertility [127]. Hence, ROS are an essential part of the spermatozoa function, although excessive ROS levels are detrimental to fertility. In a male mice model, oxidative stress was also associated with DNA damage in both testicular and epididymal sperm [125], partially driven by increased activity of testicular macrophages (but not necessarily their numbers) [128]. Obesity is also responsible for increasing testicular apoptosis, both in spermatozoa [126,129] (although only [126] made a clear connection between apoptosis and oxidative stress) and germ cells [122]. In both cell types, caspases 9, caspase 3 and cytochrome c are involved in cell death, indicating a preference towards the intrinsic pathway of apoptosis [122,126]. Increased apoptosis likely contributes to low sperm counts. The reduced motility described is most likely due to cell membrane lipid peroxidation and its lipid aldehyde products 4-hydroxynonenal and acrolein inhibiting motility [130], which causes loss of membrane fluidity and integrity [131]. All these negative effects are also potentiated by the lack of testosterone. The decline in androgen levels causes loss of integrity in the blood–testis barrier [132], halts spermatogenesis at the conversion of round spermatids from stage VII to stage VIII [133], prompts premature detachment of these spermatids from Sertoli cells [134], and lowers LDH expression in Sertoli cells [135].

Adipokines, particularly adiponectin and leptin, also influence semen parameters. On one hand, adiponectin increases antioxidant enzymes (SOD, CAT, GPx), glucose uptake and LDH activity in Sertoli cells, and decreases germ cell apoptosis [118]. These effects justify its protective role on sperm motility and nuclear DNA integrity [83]. We can hypothesize that it improves (or at least does not hinder) sperm counts, since it improves Sertoli cell metabolism and reduces germ cell death. Alternatively, leptin holds positive effects when in physiological concentrations (enhancing the activity of GLUT2 and LDH [136], upregulating steroidogenic enzymes [137], increasing the number of seminiferous tubules engaged in the first stages of spermatogenesis [137]) and negative effects when in supraphysiological concentrations—as is the case in obesity (reducing sperm progressive motility [138] and count [139], increasing DNA fragmentation [140], changing the structure of the seminiferous tubules [139]). Since obesity reduces adiponectin and increases leptin beyond physiological levels, the positive effects of these adipokines are replaced by detrimental effects, such as the reduction in most sperm quality parameters.

At a histological level, oxidative stress induced by immobilization stress caused seminiferous tubular atrophy and germ cell shedding due to low testosterone levels [141], which is similar to what is detected in HFD-fed male C57BL/6 mice (12 weeks), with decreased seminiferous tubules diameter and height, increased lumen diameter, and low overall testis weight [142]. Similar results were found in HFD-fed male Sprague-Dawley rats (12 weeks), also with reduced germ cell and spermatozoa populations [83]. Osváth and colleagues found that oxidative stress disturbs vascular tone and reduces testicular blood flow, regardless of the cause [143]. In accordance, the same was found in C57BL/6 mice with obesity [144]. Leydig cells may also be affected by obesity not only at the functional level. The obesity-mediated increase in estrogen (especially estradiol) favors Leydig cell phagocytosis by macrophages activated by the estrogen receptor α [145,146]. This could result in a decline in the Leydig cell population, further aggravating this estrogen-favoring cycle. Interestingly, a study on young adult male monozygotic twin pairs, where one twin was overweight, showed that dihydrotestosterone (an androgen 10x stronger than testosterone [147]) and sex hormone-binding globulin (SHBG—a protein that transports sex steroids in blood [148]) are significantly reduced in the co-twin with the highest weight [149]. The same study highlighted that the greater AKR1C2 expression in the adipose tissue of the twins with obesity was responsible for inactivating dihydrotestosterone (converted to androgen 3α-diol) [149].

The described systemic and testicular effects impair the ability of the prospective father to conceive. However, if the father has successfully conceived, would there be any consequences to his descendants? The short answer seems to be yes. The work published by Crisóstomo and colleagues demonstrated in C57BL6/J mice (HFD-fed for 200 days, weaning at day 120) that obesity promotes intergenerational (father-to-son) and transgenerational (grandfather-to-grandson) impairments to the reproductive health of the lineage from the individual with obesity. This includes reduced testicular weight (intergenerational), increased body weight at weaning (intergenerational), reduced sperm count and normal morphology (transgenerational) and an increase in testicular acetate (transgenerational). This indicates that intergenerational changes are associated with altered testicular biometric characteristics, whereas transgenerational changes are associated with altered sperm parameters [150]. The first generation after HFD exposure (intergenerational) showed alteration in testicular amino acid content, namely high levels of testicular glycine and leucine [150], while the second generation (transgenerational) showed alterations in testicular fatty acid content, including the increase in acetate, ethanolamine, and oleic acid, and the decrease in phosphocholine, phosphoethanolamine, docosapentaenoic acid (ω3 and ω6), docosahexaenoic acid and polyunsaturated fatty acids (ω3 and ω6), among others [151]. There was also a significant differential epigenetic fingerprint in sperm small non-coding RNA (sncRNA), with the first generation after HFD-exposure (intergenerational) having two micro RNAs (miRNAs), one transcription initiation RNAs (tiRNAs), 18 piwi-interacting RNA (piRNAs) and 80 repeat-derived small RNA (repRNAs) differently expressed in HFD compared to the control, whereas the second generation (transgenerational) had no significantly different sncRNA expression [152]. These sncRNAs are involved in epigenetic inheritance mechanisms and might be future targets for investigating the consequences of obesity in testicular health. These same studies proved that estradiol was increased in the second generation, even though it remained unchanged in the original and first generations [151]. While findings regarding estradiol are often statistically significant, it is crucial to interpret them within the context of each study’s methodology. Since estradiol levels can vary, it is important to note that reported values sometimes exceed the detection limit of the radioimmunoassay. Thus, variations in assay sensitivity and methodology between studies should be considered when comparing results and drawing broader conclusions [153]. Finally, these transgenerational studies established that obesity-induced compromise of testicular antioxidant defenses are transversal to all three generations, especially regarding glutathione-disulfide reductase (GSR) (intergenerational) and CAT levels (inter and transgenerational) [151]. These findings help explain the observed increase in sperm ROS following HFD-induced obesity, which promotes DNA damage and reduces sperm motility, as described by Fullston and colleagues [154]. Although there is consensus that testicular oxidative defenses are disrupted across generations, this sperm motility reduction is not consistent across the limited number of transgenerational obesity studies, as Crisostomo and colleagues only observed this effect in the exposed paternal generation [150]. While significant progress has been made, these findings underscore that much remains to be explored to fully understand the complexities of the transgenerational effects of obesity and their implications on reproductive health.

**Figure 2:**
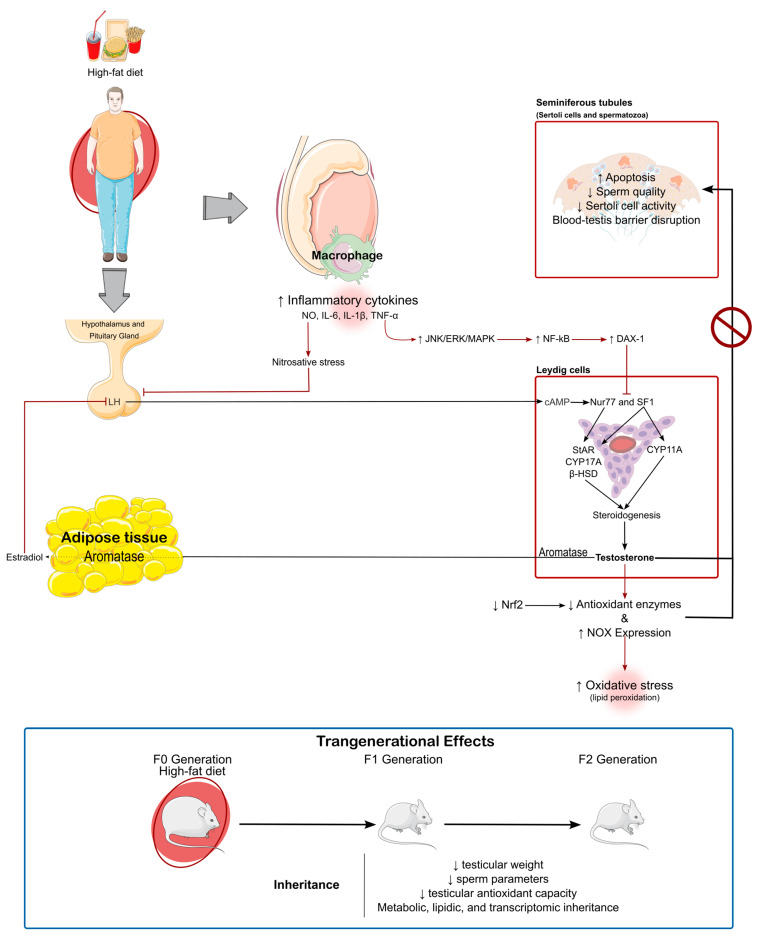
High-fat-diet-induced testicular dysfunction and its transgenerational effects. A high-fat diet induces adipose tissue hypertrophy and aromatase activity, increasing estradiol levels. Nitric oxide (NO) released by macrophages and adipose tissue contributes to nitrosative stress, impairing the hypothalamus–pituitary axis and disrupting LH signaling. Other inflammatory cytokines, such as Tumor Necrosis Factor-alpha (TNF-α), Interleukin-6 (IL-6), Interleukin-1β (IL-1β), exacerbate inflammation, further impairing Leydig cell function. Leydig cells exhibit reduced steroidogenic activity due to the activation of the JNK/ERK/MAPK pathway that culminates in the increased Nuclear Factor Kappa B (NF-κB) and DAX-1, which inhibits Nur77 and steroidogenic factor 1 (SF1), transcription factors involved in the expression of steroidogenic enzymes (steroidogenic acute regulatory protein—StAR; Cytochromes P450 CYP11a and CYP17a; and β-Hydroxysteroid dehydrogenases—β-HSD, resulting in decreased testosterone production. The seminiferous tubules show increased apoptosis, disrupted Sertoli cell activity, and compromised blood–testis barrier integrity, contributing to poor sperm quality. Elevated oxidative stress, driven by reduced nuclear factor erythroid 2-related factor 2 (Nrf2)-mediated antioxidant defenses and increased NADPH oxidase (NOX) activity, exacerbates lipid peroxidation and testicular damage. Transgenerational effects observed in the F1 and F2 generations include reduced testicular weight, decreased sperm parameters, and impaired antioxidant capacity, accompanied by metabolic, lipidic, and transcriptomic inheritance. Red arrows represent the negative effects induced (regular arrow—effect induced; inhibition arrow—process inhibition). ↓—downregulation. ↑—upregulation. Red box—negative effects. Blue box—transgenerational effects.

Obesity-induced oxidative stress acts as a multifactorial player in the impairment of spermatogenesis and steroidogenesis. It creates a dyad with inflammation, where one fuels the other and both inflict damage in the testes when left unchecked. Obesity is also associated with shifts in the individuals’ endocrine profile, with high leptin and low adiponectin coming from the expanding adipose tissue. This shift is also present when considering the sex steroids testosterone and estradiol, which decrease and increase obesity, respectively. The low testosterone is caused by altered testicular blood flow, damage and halted activity of Leydig cells, unbalanced oxidant/antioxidant status, adipose aromatase activity, hyperleptinemia and hypoadiponectinemia. The decline in testosterone, along with direct oxidative stress prompted by ROS, compromises seminiferous tubule integrity and sperm cell development. Some of these effects can be perpetuated across (at least) two generations. Even if oxidative stress is not the sole cause of obesity-driven testicular damage, it is central in its pathophysiology; thus, broad approaches that target oxidative stress (along with other aspects of testicular pathophysiology of obesity) should be considered. Figure 2 illustrates the current understanding of high-fat diet-induced testicular dysfunction and its transgenerational effects as documented in the literature.

## 4. Improving Testicular Health by Reducing Oxidative Damage Through Lifestyle Choices

Weight loss strategies are fundamental to address obesity and its associated comorbidities, such as type 2 diabetes mellitus, dyslipidemia, hypertension, infertility and others [155]. Numerous interventions are available to enable weight loss, such as bariatric surgery [156,157] and pharmacological therapies, for instance, incretins such as glucagon-like peptide-1 (GLP-1) analogs [158] or metformin [159], among others. However, these interventions are not without limitations and carry potential risks.

Bariatric surgery, while highly effective for significant weight loss, is invasive and accompanied by the possibility of surgical complications [160]. Pharmacological options, although less invasive, are often associated with side effects. The GLP-1 analogs, for instance, may cause nausea, vomiting, diarrhea or constipation [161]. Metformin may cause similar side effects, as well as lactic acidosis, and hepatotoxicity. Since these therapies are associated with pronounced weight loss, there is a risk of nutritional imbalance, which in turn can result in toxicant accumulation [162]. Another concern is weight cycling—also known as yo-yo dieting, which refers to the repeated loss and regaining of body weight. The consecutive loss and gain of weight is detrimental to health and may lead to the development of conditions associated with metabolic syndrome [163,164].

Considering these limitations, lifestyle improvements for progressive weight loss are the first line of treatment for obesity before considering pharmacological or surgical interventions. In this chapter, we will discuss the key aspects of lifestyle-based approaches—dietary correction and physical activity—as effective means of reversing obesity-induced weight gain and mitigating oxidative damage to the testis.

### 4.1. Nutritional Approaches Through Caloric Restriction

Caloric restriction, a dietary intervention characterized by reduced energy intake without compromising adequate nutrition, is widely recognized for its ability to extend a healthy lifespan and to ameliorate metabolic and hormonal pathologies [165]. Its effects on obesity have been extensively researched with a generalized consensus that it reduces BMI in human [166,167] and animal studies [168,169].

Regarding the hormonal profile, caloric restriction increases serum testosterone in humans (low-fat diet—LFD: 800 kcal/d for 12 weeks) [167] and mice (LFD: 9.35% kcal from fat for 8 weeks) [169], as well as serum adiponectin in humans (very LFD: 654 kcal/d for 6 weeks alternated with a personalized LFD for a total of 24 weeks) [166] (low-fat diet—LFD: 800 kcal/d for 12 weeks) [167]. Concurrently, it reduces serum leptin and insulin in humans [167] and rats (30% food consumption reduction from control for 28 days) [168]. These alterations clearly indicate that caloric restriction shifts the hormonal profile away from the patterns associated with obesity and metabolic syndrome.

Regarding reproductive health, mice that underwent caloric restriction evidenced a higher testicular coefficient [(weight of both testes/body weight) × 100)] compared to mice with obesity (25% food consumption reduction from HFD for 12 weeks) [169]. The same study showed that low calory intake improves several sperm parameters such as sperm count, motility and morphology compared to obesity. However, two separate studies in rats reported that caloric restriction increases the number of head abnormalities in spermatozoa when compared to standard-diet-fed lean rats (30% food consumption reduction from control for 28 days) [168,170]. This is the main side effect reported of male fertility; however, overall spermatogenesis is increased by caloric restriction (25% food consumption reduction from HFD for 12 weeks) [169]. At the systemic level, caloric restriction bolsters antioxidant defenses. Serum levels and activity of several antioxidant enzymes (GSH, GSR, SOD, CAT) are upregulated in humans (very LFD: 654 kcal/d for 6 weeks alternated with a personalized LFD for a total of 24 weeks) [166] (300–500 kcal/day reduction for 8 weeks) [171] and mice (25% food consumption reduction from HFD for 12 weeks) [169]. This results in reduced testicular oxidative stress, particularly lipid peroxidation (30% food consumption reduction from control for 28 days) [168] (25% food consumption reduction from HFD for 12 weeks) [169]. Further contributing to this effect are reductions in serum myeloperoxidase activity (300–500 kcal/day reduction for 8 weeks) [171], lower levels of PAI-1 (25% food consumption reduction from HFD for 12 weeks) [169] and circulating polymorphonuclear leukocytes (300–500 kcal/day reduction for 8 weeks) [171], as well as increased testicular gene expression of FTO, MC4R, GNPDA2, TMEM18 (30% food consumption reduction from control for 28 days) [170] and SIRT1 (25% food consumption reduction from HFD for 12 weeks) [169]. FTO, MC4R [170] and SIRT1 [169] are responsible for increasing the expression of Nrf2, favoring the antioxidant response [172]. TMEM18 is a key gene involved in maintaining sperm viability [170]. GNPDA2 is responsible for reducing insulin resistance; however, the authors hypothesized that the metabolic alterations prompted by this gene caused the head abnormal morphology registered [170].

Dietary supplementation may also be considered to complement the benefits of dietary correction for individuals with obesity. A meta-analysis from 2022 showed that antioxidant supplements drive positive effects in this population, prompting a reduction in BMI, fasting blood glucose levels, and MDA, as well as an increase in serum SOD levels [173]. Resveratrol is an example of a potential dietary supplement that increases sperm parameters (progressive motility and morphology) in mice [174]. Another potential candidate is sulforaphane, which increases the antioxidant response in mice with obesity through Nrf2 upregulation and activation [175]. In rats, vitamin E has also a considerable effect in mitigating impairments induced by obesity in the testis, mostly by reversing damages in Leydig cells and stimulating steroidogenesis [176]. Melatonin has also been studied in this regard, presenting positive effects against obesity, such as an increase in testosterone production, testicular SOD, sperm count and motility; a reduction in testicular apoptosis; and reversion of histomorphometrical alterations [177]. There are strong indicators that the use of these supplements is positive to reverse fertility struggles caused by obesity; however, more work is needed to find the optimal conditions in which these can be consumed and if these could be used in a preventive manner, instead of damage control.

The study from Crisóstomo and colleagues that proved that an obesity-induced testicular phenotype in mice can be inherited for up to two generations also demonstrated that transitioning from an HFD to a calory-restricted diet protects against this obesity-induced inheritable impairment (reduction from 36.0–59.0% fat to 7.2–16.3% for 80 days) [150]. In particular, diet transition restored testicular weight and body weight at weaning to group levels (intergenerational), reduced testicular acetate levels (transgenerational), leucine (intergenerational), and increased the number of sperm cells with normal morphology (transgenerational) [150]. However, it was not able to reverse transgenerational reduction in sperm count [150]. Regarding the alterations in the testicular lipidome, diet transition reduced acetate, ethanolamine, and oleic acid (transgenerational), and increased phosphocholine, phosphoethanolamine, docosapentaenoic acid (ω3 and ω6), docosahexaenoic acid, and other ω3 and ω6 polyunsaturated fatty acids (transgenerational) [151]. Finally, regarding testicular RNA content, diet transition changes one miRNA, four tRNA-derived fragments (tRFs), two tiRNAs, three piRNAs, one repRNA in the first generation (intergenerational) and six miRNAs in the second generation (transgenerational) compared to the HFD-exposed groups [152]. Contrary to the HFD-fed mice, the grandsons of mice submitted to diet transition had estradiol at control levels, showing that diet transition improves testosterone availability by reducing aromatization [151]. Finally, diet correction improved the GSR and CAT activity in the first generation after exposure to HFD (intergenerational) but was not able to do the same for the second generation. These studies clearly demonstrate that not only do obesity-induced impairments have a hereditary component, but also that diet transition to caloric restriction reverts some of those effects, potentially protecting not only the exposed generation but their offspring as well. However, more research is necessary to ensure the safety and effectiveness of this type of intervention. Particularly considering that Anuradha and colleagues recently described that excessive caloric restriction (50% caloric restriction for 8 weeks) caused paternal undernutrition in rats. In turn, this deficient nutrition decreased the health and longevity of the offspring, mostly by reducing the adiponectin and SIRT1 expression and causing inflammation, while the father boasted increased expression of both and reduced inflammation [178].

**Figure 3:**
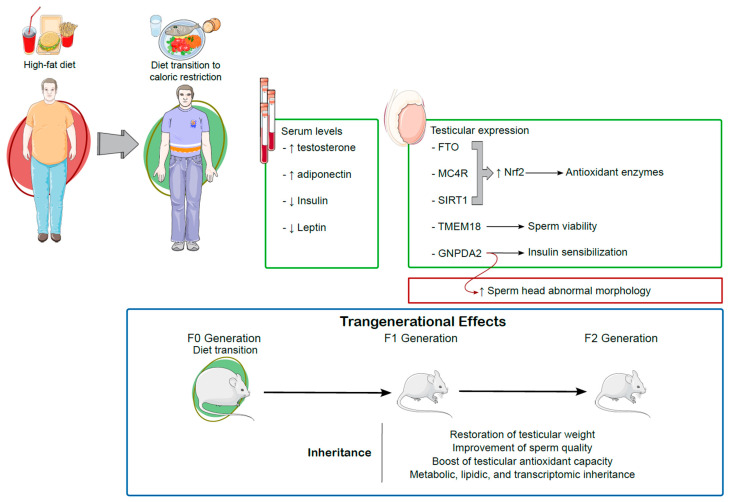
Effects of dietary transition from a high-fat diet to caloric restriction on male reproductive health and transgenerational outcomes. Transitioning from a high-fat diet to caloric restriction leads to improvements in serum markers, including increased testosterone and adiponectin levels, and reduced insulin and leptin levels. In the testes, enhanced expression of key genes—fat mass and obesity-associated protein (FTO), melanocortin 4 receptor (MC4R), sirtuin 1 (SIRT1), glucosamine-6-phosphate deaminase 2 (GNPDA2), transmembrane protein 18 (TMEM18)—promotes antioxidant enzyme activity (via Nrf2), sperm viability, and insulin sensitization. However, caloric restriction is associated with an increase in abnormal sperm head morphology. Transgenerational benefits observed in the F1 and F2 generations include restored testicular weight, improved sperm quality, increased testicular antioxidant capacity, and enhanced metabolic, lipidic, and transcriptomic profiles, suggesting intergenerational inheritance of positive dietary effects. ↓—decrease. ↑—increase. Green box—positive effects. Red box—negative effects. Blue box—transgenerational effects.

Caloric restriction is a nutritional strategy to counteract obesity-induced testicular impairments, offering systemic and reproductive benefits. By restoring hormonal balance, reducing oxidative stress, and enhancing sperm quality, caloric restriction not only addresses immediate metabolic and reproductive dysfunctions but also mitigates the transgenerational inheritance of these impairments. Dietary supplementation further amplifies these benefits, with specific antioxidants showing the potential to reverse or prevent testicular damage. However, caution is needed to optimize caloric restriction protocols and supplementation strategies, ensuring the minimization of side effects such as sperm morphological abnormalities. Overall, these findings highlight the potential of nutritional interventions as first-line, non-invasive strategies to protect and restore male reproductive health in the context of obesity. They also reinforce the importance of addressing lifestyle factors early to prevent the long-term and hereditary consequences of obesity on testicular function. Figure 3 provides a comprehensive summary of the current literature on the effects of dietary transition from a high-fat diet to caloric restriction on male reproductive health and transgenerational outcomes.

### 4.2. Physical Exercise

Physical exercise is a subset of physical activity. While physical activity encompasses any body movement that requires energy expenditure, physical exercise is planned, structured, and performed with the explicit aim of improving or maintaining components of physical fitness [179]. The role of physical exercise in promoting a healthy lifestyle is well-established. It is widely accepted that physical exercise is a crucial factor in maintaining a healthy lifestyle, since it improves quality of life and (physical and mental) health outcomes [180], reduces mortality and cardiovascular events [181], prevents atherosclerotic cardiovascular disease [182], enhances inspiratory muscle strength and endurance [183], improves the outcomes in cases of tumors (namely breast tumor) [184], among other effects. As might be expected, physical exercise also exerts effects on the male reproductive tract, influencing overall reproductive health. These effects are summarized in Table 1.

Physical activity exerts a variety of influences on testicular structure, function, and overall reproductive health. The outcomes vary significantly depending on the intensity, duration, and type of exercise, as well as the species studied. On a macroscopic level, wheel physical activity reduces testicular and seminal vesicle weight in mice [197]. On a histological level, it reduces the number of spermatogonia, presumably due to the increased commitment of these precursor cells to form spermatids, which are increased after running in mice [197]. However, when it comes to physical exercise, this is not always the case, considering that two studies using Wistar rats proved that high-intensity treadmill exercise reduced spermatogenesis (as assessed by Johnsen’s score) [191], spermiogenesis [192], and seminiferous epithelium height [191,192].

Sperm parameters are also influenced by physical exercise, with sperm concentration being increased by (undisclosed) physical exercise [187,198] and by moderate resistance exercise [186] in humans and reduced by high-intensity running in rats [191] and by high-intensity bicycling [188] and triathlon (professionalized high-intensity exercise) in humans [185,189]. Sperm motility is increased by running exercise in mice [193] and moderate resistance exercise in humans [186], and reduced by bicycling in humans [188] and triathlon [189]. Sperm morphology and DNA fragmentation are increased by moderate resistance exercise [186] and reduced by triathlon [185] in humans. The triathlon results remain ambiguous since it was unclear whether the results acquired from triathletes were induced by the bicycling component of the sport or by the combination of high-intensity running, bicycling and swimming.

Regarding the main testicular somatic cells, Sertoli and Leydig cells are both increased in number in mice after lifelong physical activity compared to sedentary mice [197]. Even so, the hormones that stimulate these cells, FSH and LH, respectively, are decreased in rats after intensive swimming exercise [195]. These low hormonal levels will be translated into lower activity from these cells. This is particularly evident when assessing LDH activity and testosterone production. LDH, a critical enzyme for Sertoli cell function and its expression, as well as activity, is reduced in rats that followed a high-intensity exercise protocol, diminishing the germ cell substrate (lactate) [192]. In the case of Leydig cells steroidogenesis, serum testosterone levels are reduced in rats after intensive swimming [195,196] and running [190,194] regimens. High-intensity physical exercise appears to disturb testosterone production, yet in moderate intensities in mice, it does the opposite, promoting significant testosterone production [193]. 

Overall oxidative and nitrosative stress is reduced by physical activity (wheel exercise; mice) [197] and worsened by high-intensity exercise (treadmill; rats) [191,192]. This is a pattern that is reflected across several indicators of testicular redox status, with physical activity and moderate-intensity exercise being more beneficial than high-intensity exercise. Lipid peroxidation is decreased by wheel exercise in mice [197] and by moderate-intensity resistance exercise in humans [186], and increased by intense swimming [195,196] and intense running [191] in rats. Antioxidant enzymes, such as SOD, CAT, and GST, are increased by moderate-intensity resistance exercise in humans [186] and reduced by intensive swimming in rats [195,196]. This oxidative stress results in high intensity and is the most likely cause of Sertoli and Leydig cell dysfunction, by reducing LDH activity and expression of steroidogenic enzymes, and the altered sperm parameters and DNA fragmentation.

High-intensity running also prompts testicular apoptosis and autophagy in mice. This mitochondria-mediated apoptosis, with increased expression of Bax and caspase 3 (whereas low-intensity exercise upregulates Bcl-2 expression) is mostly caused by oxidative stress and lack of antioxidant response [191]. Intense running-induced autophagy is also present in mice testis, because unlike other exercise intensities, mice that perform high-intensity exercise do not eliminate autophagy-inducing molecules, leading to their accumulation [192].

Inflammation follows the same pattern as oxidative stress, with moderate-intensity resistance exercise in humans reducing seminal IL-1β, IL-6, IL-8 and TNF-α [186] and high-intensity running in rats increasing HIF-1α and TNF-α [192]. In humans, high-intensity sports, such as triathlons, cause testicular infiltration and activation of macrophages [185], which are detrimental to fertility, as previously addressed. Nazanin and colleagues showed that a Wistar rat model exposed to low-, moderate- and high-intensity treadmill exercise had increased IL-10 only in low intensity, increased TLR-4, NF-κB, IL-6, iNOS and COX-2 in moderate and high intensity (with all of these effects being more pronounced in high intensity). However, the negative effects on testosterone (low serum testosterone and low active Leydig cells) were only present in rats with high-intensity regimens [190]. Although these differences can arise from protocolar differences, such as the time of sample collection after exercise, we can hypothesize that low-intensity exercise does not cause any inflammation, and while both moderate and high-intensity exercises do cause inflammation, the levels in moderate exercise are less detrimental and the organism has compensatory mechanisms to tolerate this inflammation, which may not be the case in high-intensity exercise. Further research is needed to test this hypothesis.

For a long time, physical exercise has been the “nemesis” of obesity by mitigating or reverting its most distinctive feature—weight gain by fat accumulation [199,200]. Thus, it is understandable that the effects physical exercise boasts on obesity have repercussions throughout the body, as is the case in the testis. These effects are summarized in Table 2.

In mice with obesity, treadmill running significantly improves the testis weight/body weight ratio [142]. This is accompanied by increased seminiferous tubule diameter and epithelium height (and consequent lumen reduction) [142]. A similar effect is observed in rats with obesity, where a moderate swimming protocol reduced testicular atrophy [206]. These findings suggest that the positive effects of exercise may not be solely dependent on the type of exercise but rather on its intensity.

As previously discussed, obesity induces several dysfunctions in sperm parameters, and it appears that physical exercise can counteract these effects. Xu and colleagues reported that treadmill exercise in mice restored sperm concentration and motility to levels comparable to the control group [142], while Nematollahi and colleagues reported that the same exercise type in the same animal model increased sperm concentration and motility beyond that of the control [209]. Differences in protocols may explain these discrepancies; however, Rosety and colleagues also described an increase in these sperm parameters that exceeded the control group in humans submitted to a treadmill exercise regimen [203]. Also in humans, Kumagai and colleagues showed in a longitudinal study that after 12 weeks of walking and/or light jogging, sperm concentration and motility are significantly increased compared to the analysis before the 12-week period [202]. Studies focusing on exercise intensities have consistently shown that moderate-intensity physical exercise is more effective in enhancing sperm concentration and motility, whereas high-intensity exercise paradoxically reduces these parameters to levels comparable to those in obesity [28,210].

Obesity-induced increases in sperm mitochondrial membrane potential—a factor contributing to low motility—are mitigated by exercise, although not fully reversed, as values remain higher than in control groups [212]. Moderate-intensity swimming has been shown to enhance the percentage of sperm with normal morphology in rats [208]; however, this increase was concurrent with the increase in abnormal tail morphology in both rats [208] and mice [212]. Yet, in the previously mentioned study by Rosety and colleagues sperm normal morphology was higher after a treadmill exercise protocol compared to control [203], while in the study by Kumagai and colleagues, it increased after walking/jogging compared to when the protocol began [202]. In addition to improving sperm parameters, physical exercise has demonstrated the ability to reverse obesity-induced sperm damage, restoring healthier sperm function. In particular, it restores protamine levels in sperm [209] and germ cell DNA damage [207], which are interrelated, as insufficient sperm protamine levels make DNA more vulnerable to damage, with both factors being associated with reduced fertility rates [213].

Regarding spermatogenesis (as well as spermiogenesis), it is increased in rats after moderate swimming exercise [206] and treadmill exercise [207] when compared to rats with obesity. This is attributed to increased serum FSH levels (in mice [142] and rats after a treadmill regimen [205], and in rats after a swimming regimen [206]). Another reason is the increase in the number of Sertoli cells in the seminiferous tubule (rats submitted to a treadmill running regimen) [207]. Beyond cell numbers, Sertoli cells are also more functional, with higher LHD activity and higher expression of GLUT-1 and GLUT-3 (allowing more glucose uptake), Igf1 (upregulating lactate production), and MCT-4 (favoring the transport of lactate to germ cells). These alterations from the obese phenotype favor lactate production and uptake by germ cells and are especially evident in high-intensity exercise protocols [204]. Germ cell number is also increased in rats that followed this exercise regimen compared to rats with obesity [207], showing their susceptibility to substrate limitation.

Much like in spermatogenesis, steroidogenesis is also affected by obesity, raising interest in the effects of physical exercise in this important process. In humans, serum testosterone levels increase significantly post-exercise [202,203], with resistance exercise showing greater efficacy than aerobic exercise [201]. Another study in humans showed that testosterone and sex hormone-binding globulin (SHBG) are increased after 14 weeks of physical exercise; however, the regimen and intensity are undisclosed [198]. Animal studies reveal mixed results: You and colleagues and Azar and colleagues used two different strains of rats that were subjected to treadmill exercise and concluded that testosterone is increased by this exercise regimen; however, the first reported that exercise was enough to change testosterone to control levels [211], whereas the second reported that this regimen increased testosterone above the levels found in rats with obesity, but still lower than the lean control [207]. In mice, Yi and colleagues found that in mice only moderate-intensity treadmill exercise increases testosterone to control levels and that high-intensity protocols maintain this androgen in obesity levels [28,210]. These results are in accordance with the one from Table 1; however, Xu and colleagues attributed this positive effect only to high-intensity treadmill exercise [142]. Variability in these findings may stem from differences in exercise protocols and animal strains. Nevertheless, there seems to be consensus that exercise improves steroidogenesis. This may come from the increase in serum LH [205,206] and from the increase in Leydig cell number [207] and function. After swimming and treadmill exercise in rodents, there is an increase in Leydig cell function since StAR, CYP11A1, CYP17A1, and SF-1 are more expressed compared to obesity groups [28,142,210].

Aerobic moderate-intensity exercise is associated with increased total antioxidant status and decreased oxidant status [28,205]. The shift in these statuses is a result of the increase in antioxidant enzymes, such as SOD, CAT, and GPx [28]. Physical activity, such as voluntary wheel exercise in male rats, can modulate a key mechanism regarding antioxidant enhancement, which involves the downregulation of miR-34a, leading to increased SIRT1 expression, which activates Nrf2 and promotes antioxidant defense [214]. This microRNA reduces the translation of SIRT1 mRNA, thus reducing the levels of this protein. When present, SIRT1 is responsible for activating Nrf2 by deacetylation [172], which promotes the expression of antioxidants such as HO-1 and NQO [142]. The relation between SIRT1 and Nrf2 reduces oxidative stress in Leydig cells, favoring steroidogenesis [123] and in germ cells, favoring spermatogenesis [215]. A key mechanism involves the downregulation of miR-34a, leading to increased SIRT1 expression, which activates Nrf2 and promotes antioxidant defense. For instance, while high-intensity treadmill exercise reduces lipid peroxidation [142], moderate-intensity swimming appears more effective [28], though levels often remain elevated compared to controls [214].

As would be expected, physical exercise also reduces overall obesity-induced apoptosis in the seminiferous tubules [142,206]. Sperm apoptosis is decreased as well; however, this is only the case for low- to moderate-intensity exercise [28,207]. Despite this decrease, apoptosis is still higher in exercised animals with obesity compared to control [207], indicating that moderate physical exercise alone is insufficient, warranting the addition of complementary interventions to reduce testicular apoptosis. The mechanisms involved in this apoptosis inhibition are still not fully understood, given that most studies focus on broader parameters of apoptosis, such as sperm apoptosis rate [28] and testicular or tubular apoptotic indexes [206,207,214], instead of particular apoptotic pathways. Only Xu and colleagues attempted to demonstrate which pathway was linked to this reduction, concluding that it was most likely the intrinsic pathway, given that Bax and Bcl-2 were involved [142], but no explanation was given regarding how exercise modulates this pathway. Nevertheless, this represents a positive effect of exercise against obesity, as it is a reason behind the reported increase in sperm count of exercised subjects.

The persistent low-grade inflammation associated with obesity is a critical aspect of the positive effects boasted by exercise in subjects with obesity. In rats and mice submitted to treadmill exercise or swimming exercise the serum levels of inflammatory cytokines such as TNF-α, IL-6 [206], IL-1β, NO [28], and MCP-1 [211]. This effect was observed with moderate-intensity exercise, whereas high-intensity exercise maintained cytokine levels in the exercise groups similar to those in the obesity group [28]. These effects mainly result from the reduced expression of NF-κB [28]; however, the upstream mechanism is not clarified.

All the positive effects in spermatogenesis and steroidogenesis are also supported by a decrease in inflammation and oxidative stress, as well as alterations in the endocrine profile. Obesity promotes hyperinsulinemia, hyperleptinemia and hypoadiponectinemia. These hormonal alterations act as systemic modulators of the damages caused by obesity, exacerbating metabolic dysfunction and contributing to inflammation and oxidative stress. Physical exercise normalizes these levels by reducing serum insulin, insulin resistance, and fasting glycemia [201,202]; lowering serum leptin [206,210] and leptin resistance [211]; and by increasing serum adiponectin [216]. All these alterations work in tandem to improve testicular function to non-obesity standards.

The combination of physical exercise and dietary interventions seems to have a positive outcome on men’s reproductive health, with the combination of caloric restriction and exercise (aerobic or resistance) appearing as the most favorable combination to improve and maintain body mass [217]. A 13-week randomized control trial showed that combining these lifestyle modifications could improve circulating adipokine and anti-inflammatory cytokine levels [218]. However, much is still to uncover when it comes to this lifestyle combination, especially in men.

As previously outlined, the effects of physical exercise on reproductive health are intensity-dependent, with moderate and high-intensity exercises yielding distinct outcomes. In humans, moderate-intensity weightlifting exercise enhances testicular function by exerting anti-inflammatory (reduction in seminal IL-1β, IL-6, IL-8 and TNF-α) and anti-oxidant effects (reduction in seminal ROS, and lipid peroxidation and increase in SOD and CAT activity) [186]. These changes coincide with sperm quality improvement. Conversely, high-intensity physical exercise, especially triathlon, reduces some of these parameters and promotes the infiltration of macrophages in the testis [185,189]. In rodents, intensive swimming exercise promotes testicular oxidative stress and suppresses Leydig cell steroidogenesis [195,196]. Comparative studies on the effects of physical exercise and obesity in rodents highlight the benefits of moderate-intensity exercise. It improves Sertoli cell activity [204], increases testosterone production [28,210], potentiates antioxidant defenses [28], and reduces germ cell DNA fragmentation [207]. Conversely, high-intensity exercise can lead to testicular dysfunction, mimicking some aspects of the obesity phenotype—reduced sperm motility, count, serum testosterone and steroidogenic enzymes [28], and the increase in oxidative stress, inflammation, and sperm apoptosis [28,191]. Interestingly, another study showed favorable outcomes from high-intensity exercise, such as increased serum FSH and testosterone and reduced testicular oxidative stress through the Nfr2 pathway [142]. These discrepancies may arise from experimental differences. For instance, the study reporting a significant negative effect of high-intensity exercise used swimming exercise (20 min/day, twice a day, 5 days/8 weeks) [28], while the one that showed more favorable results employed treadmill exercise (HIEP: 70−75% of Smax, 45 min/day, 5 days/8 weeks) [142]. Despite this fact, a precedent emerges suggesting potential adverse effects of high-intensity exercise on reproductive health in both humans and rodents. Further research is needed to optimize high-intensity exercise regimens and tailor them for personalized approaches that maximize benefits while minimizing risks. The inter and transgenerational effect of exercise against obesity is not as well studied as the effect of diet transition of caloric restriction. While some work has been carried out on the effect of maternal exercise on intergenerational obesity [219,220], it is limited and warrants further investigation. Regarding the paternal influence, even less work is available. However, an epigenetic study showed some promise, indicating that paternal exercise could improve the metabolic health of the offspring [221]. Despite this, a study on long-term physical exercise regimens indicates that these interventions could reprogram the offspring towards a phenotype of increased susceptibility to obesity due to low energy expenditure [222], suggesting a possible negative transgenerational effect of physical exercise. It would also be of interest not only to clarify these effects but also to assess the transgenerational benefits of combining different lifestyle modifications, since there is no significant work in the literature in this regard.

**Figure 4:**
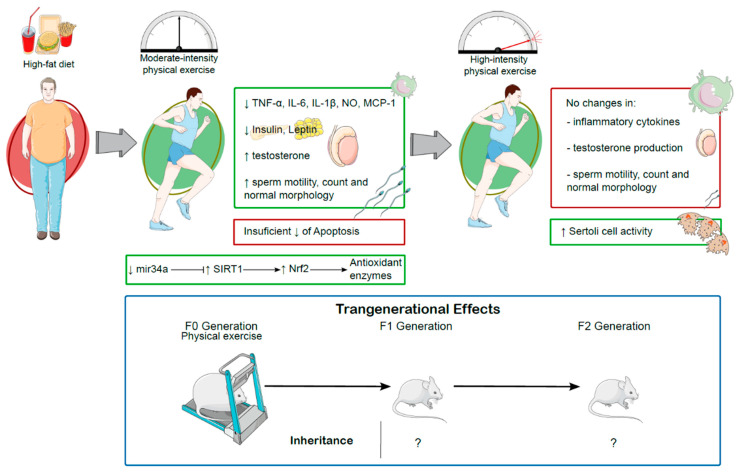
Effects of physical exercise on male reproductive health and potential transgenerational effects. Moderate-intensity physical exercise decreases inflammatory cytokines (Tumor Necrosis Factor-alpha—TNF-α; Interleukin-6—IL-6; Interleukin-1β—IL-1β; nitric oxide—NO; and monocyte chemoattractant protein-1—MCP-1), insulin, and leptin levels, while increasing testosterone levels, improving sperm motility, count, and normal morphology, and resulting in insufficient apoptosis. The positive effects of moderate exercise loads are associated with decrease in microRNA mir34a, an increase in sirtuin 1 (SIRT1) and nuclear factor erythroid 2-related factor 2 (Nrf2), which is associated with higher testicular antioxidant capacity. High-intensity physical exercise shows no changes in inflammatory cytokines, testosterone production, or sperm motility, count, and normal morphology, but increases Sertoli cell activity. Transgenerational effects of physical exercise on damaged testicles remain unknown. ↓—downregulation. ↑—upregulation. Green box—positive effects. Red box—negative effects. Blue box—transgenerational effects.

Physical exercise and the associated weight loss are fundamental for reversing obesity-induced damage in the testis, by improving the metabolic profile, reducing the testicular oxidative stress and inflammation, stimulating steroidogenesis and spermatogenesis, and ameliorating sperm quality. However, more work is needed to understand what type of exercise has a favorable benefit-to-risk ratio. Considering that some exercise was not enough to reverse some of the damages induced by obesity, such as lipid peroxidation, it is important to study how it could be complemented with other approaches, such as diet correction, to maximize the health benefits of lifestyle modifications. It is also evident that different exercise intensities boast different effects, with some contradictory results in the literature. Therefore, more work should be carried out to better understand the different effects of each exercise type. The current literature on the effects of physical exercise is comprehensively summarized in Figure 4.

## 5. Long-Term Consequences of Childhood Obesity in Adult Reproductive Health and Strategies to Reverse Them

It has been established that BMI during childhood is positively correlated with adiposity during adulthood [223]. This trend is worrisome, reflecting long-term deterministic consequences of elevated childhood BMI on adult well-being and reproductive health. Obesity in boys (10–19 years old) promoted the reduction in serum SHBG and total testosterone and the increase in serum estrogen, independently of the pubertal stage [224]. Furthermore, obesity reduces serum adiponectin [225] and increases serum leptin [226]. Animal studies showed that early-life exposure to HFD prompts degeneration and apoptosis of Leydig cells, along with the inhibition of StAR and CYP11A1 expression [109,227], most likely caused by the shifts in the endocrine profile.

As verified in adults, obesity also induces non-tissue-specific oxidative and nitrosative stress in children and adolescents [10]. Nitrosative stress has been associated with lower progressive motility due to reduced mitochondrial membrane potential in spermatozoa [117]. Inflammation is also present in cases of obesity in childhood and adolescence, especially caused by cytokines such as MCP-1 [228], IL-6 [226,229], TNF-α and IL-1β [225,226]. In an animal study with Lewis rats, this inflammation was concurrent with the increase in testicular pro-inflammatory macrophages and was a cause for the established Leydig cell dysfunction [227].

Sperm parameter assessments in this population are constrained by ethical and biological factors, as their reproductive systems are not fully developed. However, an observational study by Gaskins and colleagues used a population of young men from 18–22 years studied the effects of physical activity and sedentarism (represented by hours of TV watching) and concluded that sedentarism negatively affects sperm concentration, while physical moderate-to-vigorous activity improves this parameter.

Physical activity is essential to enhance the systemic antioxidant status to prevent early in life oxidative damage across the organism [230]. The combination of different types of exercise, such as aerobic and resistance exercise, seems to be crucial to maintaining the physical health of children and adolescents with obesity. The most recent recommendations indicate that these groups should exercise at least three times or more per week for 60 min for 12 weeks or more for better health benefits [231]. Alternatively, combining physical activity with a more balanced diet is becoming a recognized approach for personalized treatment and prevention of childhood obesity and its short- and long-term consequences [232].

Obesity during childhood and adolescence seems to follow much of the trends of adult obesity, with increased systemic oxidative stress and inflammation disrupting steroidogenesis and spermatogenesis. Interestingly, nitrosative stress also has an influence on these negative effects. The combination of different types of physical activity or physical activity and proper nutrition are promising approaches against this global concern.

There is still much to uncover regarding the effects of obesity and lifestyle interventions on reproductive health. Table 3 summarizes the main pathways described in this paper, as these pathways should warrant attention in future studies.

## 6. Conclusions

The prevalence of obesity among adults and children has surged over recent decades, raising significant public health concerns. Obesity is strongly associated with various comorbidities, and its complications primarily arise from systemic and localized inflammation and oxidative stress. These processes, driven by immune cells such as macrophages, pro-inflammatory cytokines like TNF-α and IL-6, and oxidative enzymes including NADPH oxidase (NOX) and myeloperoxidase, lead to hormonal and metabolic imbalances. In the testis, these disruptions impair biomorphometric properties, reduce sperm quality, and lower testosterone levels. Alarmingly, these effects may extend beyond the individual, affecting offspring through intergenerational inheritance, thereby perpetuating reproductive and metabolic dysfunctions.

Lifestyle modifications, particularly caloric restriction and physical exercise are well-established first-line interventions to combat obesity and mitigate its effects. These strategies target key mechanisms such as the upregulation of the Nrf2-SIRT1 axis, which enhances antioxidant defenses and reduces oxidative stress. Moreover, physical exercise not only improves testicular function but also stimulates spermatogenesis and steroidogenesis by modulating inflammation and oxidative markers, while caloric restriction further supports metabolic restoration. Nonetheless, important questions remain about how to best implement these interventions to maximize their efficacy and minimize potential risks. Research is needed to refine caloric restriction protocols, evaluate the role of nutritional supplementation, identify exercise regimens with the highest benefit-to-risk ratio, and understand the synergistic effects of combining diet and exercise for maximum health benefits. It would also be relevant to assess the transgenerational effects of physical exercise, considering that the effects of dietary transition are already detailed in the literature.

However, it is important to acknowledge the limitations of the studies cited in this review. Most studies on the effects of diet and exercise interventions in obesity rely on animal models, where it is challenging to control and replicate the precise diet composition, caloric intake, and exercise regimens across experiments. Furthermore, variations in experimental conditions, including the age, sex, and genetic background of the animals, can introduce significant heterogeneity, making it difficult to generalize findings. These limitations, combined with the narrative nature of this review and the lack of systematic selection methods, may result in potential bias. Future research should prioritize standardized protocols and rigorous study designs to minimize variability and improve reproducibility.

By addressing obesity early and effectively, especially in children and adolescents, it is possible to mitigate the long-term effects on reproductive health and prevent the transmission of obesity-related damage across generations. Investing in tailored, evidence-based interventions is crucial to breaking this cycle and fostering a healthier future for individuals and their offspring.

## Figures and Tables

**Table 1:** Summary of the main studies regarding the effects of different types of exercise protocols on the testis in humans and rodents with normal body mass index (BMI).

Experimental Groups	Population/Rodent Strain	Exercise		Reference
		Type	Effects (Compared to Ctrl)	
**Human Studies**				
High-intensity/frequency exercise athletes (compared to reference values) (12 participants)	Professional athletes	Triathlon (high intensity)	↓ Semen volume ↓ Sperm number ↓ Sperm progressive motility ↑ Sperm DNA fragmentation Presence of active macrophages	[185]
**Ctrl group:** 24 wks sedentary (*n* = 208 participants) **EP group:** 24 wks MIEP (*n =* 199 participants)	Sedentary infertile at baseline	Weightlifting (major muscle groups)	↓ seminal IL-1β, IL-6, IL-8 and TNF-α ↓ seminal ROS ↓ seminal lipid peroxidation ↑ SOD and CAT activity ↑ Sperm progressive motility ↑ Sperm normal morphology ↑ Sperm concentration ↓ Sperm DNA fragmentation	[186]
**Ctrl group:** 6 months sedentary (50 participants) **EP group:** 6 months EP (*n =* 100 participants)	Sedentary men recurring to fertility clinic (some had normal spermogram (female causes)) at baseline	Intensive undisclosed exercise	↑ Semen volume ↑ Sperm concentration ↑ Sperm motility	[187]
**No regular exercise** (975 participants) **Regular exercise** (1286 participants)	Male partner of couples undergoing infertility treatment	Running/jogging, bicycling, weightlifting	↓ Sperm concentration (bicycling) ↓ Sperm motility (bicycling)	[188]
**Low exercise group:** low physical activity (16 nonprofessional participants) **High exercise group:** high-intensity/frequency exercise (professional) (14 water polo players; 15 triathletes)	Men that either practice regular physical activity or professional physical exercise	Water Polo Triathlon	↓ Sperm concentration (Triathlon > Water Polo) ↓ Sperm normal morphology (Triathlon > Water Polo)	[189]
**Rodent studies**				
**Ctrl group:** 9 wks sedentary (*n =* 6) **EP group:** 1 wk AP + 8 wks LIEP (*n =* 6), MIEP (*n =* 6) or HIEP (*n =* 6)	Wistar rats	Treadmill exercise (LIEP: 20–39% Smax, MIEP: 40–60% Smax, and HIEP: 61–84% Smax, 60 min/day, 5 days/wk, 8 wks)	↑ TLR-4 expression in SC, LC and spermatogonia (MIEP and HIEP) ↑ NF-κB expression (MIEP and HIEP) ↑ TNF-α expression (MIEP and HIEP) ↑ IL-6 (MIEP and HIEP) ↑ IL-10 (MIEP only) ↑ iNOS and NO (MIEP and HIEP) ↑ COX-2 (MIEP and HIEP) ↓ Active LC/Total LC ratio (HIEP only) ↓ Serum testosterone (HIEP only)	[190]
**Ctrl group:** 9 wks sedentary (*n =* 6) **EP group:** 1 wk AP + 8 wks LIEP (*n =* 6), MIEP (*n =* 6) or HIEP (*n =* 6)	Wistar rats	Treadmill exercise (LIEP: 20–39% Smax, MIEP: 40–60% Smax, and HIEP: 61–84% Smax, 60 min/day, 5 days/wk, 8 wks)	↓ spermatogenesis (Johnsen’s score; only HIEP) ↓ ST epithelium height (only HIEP) ↓ Sperm count (only HIEP) ↑ TOS (only HIEP) ↑ Lipid peroxidation (only HIEP) ↓ SOD, CAT, and GPx (only HIEP) ↑ Bcl-2 expression (LIEP only) ↑ Bax and caspase 3 (HIEP only) ↑ Apoptotic cells (HIEP only)	[191]
**Ctrl group:** 8 wks sedentary (*n =* 6) **EP group:** 8 wks LIEP (*n =* 6), MIEP (*n =* 6) or HIEP (*n =* 6)	Wistar rats	Treadmill exercise (LIEP: 20–39% Smax, MIEP: 40–60% Smax, and HIEP: 61–84% Smax, 60 min/day, 5 days/wk, 8wks)	Interstitial edema between ST (HIEP only) ↓ TDI and SPI (HIEP only) ↓ ST epithelium height (HIEP only) ↓ LDH activity (SC) and testicular lactate level (HIEP only) ↑ SC lipid droplets (HIEP only) ↓ testicular GSH level (HIEP only) ↑ NADP+/NADPH ratio (HIEP only) ↑ TOS and ↓ TAS (HIEP only) ↑ HIF-1α and TN-Fα (HIEP only) ↑ ROS (HIEP only) ↑ autophagy (HIEP only)	[192]
**Ctrl group:** 8 wks sedentary (*n =* 12) **EP group:** 8 wks EP (*n =* 12)	C57BL/6 mice	Treadmill exercise (25 m/min, 45 min/day, 5 days/wk, 8 wks)	↑ Sperm motility ↓ Sperm lipid peroxidation ↑ Serum testosterone	[193]
**Ctrl group:** 12 wks sedentary (*n =* 8) **EP group: 12** wks LIEP (*n =* 8), MIEP (*n =* 8) or HIEP (*n =* 8)	Wistar rats	Treadmill exercise (LIEP: 20 m/min, MIEP: 28 m/min, HIEP: 34 m/min, 60 min/day, 5 days/wk, 12 wks)	↓ total testosterone (HIEP only) ↑ plasma and adipose adiponectin (MIEP and LIEP)	[194]
**Ctrl group:** 6 wks sedentary (*n =* 6) **EP group:** 6 wks EP (*n =* 6)	Wistar rats	Intensive swimming (180 min/day, 5 days/wk, 4 wks)	↓ Testosterone (↓ 3β-HSD and 17β-HSD) ↓ Serum LH and FSH ↓ Antioxidant activity (CAT, SOD, GPx, GST)	[195]
**Ctrl group:** 4 wks sedentary (*n =* 6) **EP group:** 4 wks EP (*n =* 6)	Wistar rats	Intensive swimming (180 min/day, 5 days/wk, 4 wks)	↓ Testosterone (↓ 3β-HSD and 17β-HSD) ↓ Antioxidant activity (SOD, CAT, GST and peroxidase) ↑ Lipid peroxidation	[196]

Abbreviations: ↑—increase; ↓—decrease; 3β-HSD—3β-Hydroxysteroid dehydrogenase; 17β-HSD—17β-Hydroxysteroid dehydrogenases; AP—adaptative exercise protocol; Bax—BCL2 Associated X, Apoptosis Regulator; Bcl-2—B-cell lymphoma 2; CAT—catalase; COX-2—Cyclooxygenase-2; Ctrl—control; EP—exercise protocol; FSH—follicle stimulating hormone; GPx—glutathione peroxidase; GST—glutathione-S-transferase; HIEP—high-intensity exercise protocol; HFD—high fat diet; HIF-1α—hypoxia-inducible factor α-subunits; IL—Interleukin; iNOS—nitric oxide synthases; LDH—lactate dehydrogenase; LC—Leydig cell; LFD—low fat diet; LIEP—low-intensity exercise protocol; LH—luteinizing hormone; MIEP—moderate-intensity exercise protocol; NF-κB—factor nuclear kappa B; NO—nitric oxide; ROS—reactive oxygen species; SC—Sertoli cells; SD—standard diet; SHBG—sex hormone binding globulin; Smax—maximum speed test; ST—seminiferous tubules; SOD—superoxide dismutase; TAS—total antioxidant status; TLR-4—Toll-like receptor 4; TNF-α—Tumor Necrosis Factor-alpha; TOS—total oxidant status; TXNL4B—Thioredoxin Like 4B; wks—weeks.

**Table 2:** Summary of the main studies regarding the effects of different types of exercise protocols on testicular damage in humans and rodents with obesity.

Experimental Groups	Population/Rodent Strain	HFD/Obesity Effects	Exercise		Reference
			Type	Effects	
**Human Studies**					
**Ctrl group**: 13 wks sedentary (10 participants) **EP group**: 1 wk AP + 12 wks EP (10 participants)	Men with obesity and diabetes; 40 to 45 years old	(no results—the study compares the effect of Eps with sedentarism in men with obesity—Ctrl group is obese)	REP or AEP	↓ fasting glycemia (REP > AEP) ↓ glycated hemoglobin ↓ insulin ↓ insulin resistance (REP > AEP) ↑ testosterone (REP > AEP) ↑ FGF-21 (REP > AEP)	[201]
**Ctrl:** before 12 wks EP **EP:** after 12 wks EP (28 participants)	Men with obesity or overweight; 50.0 ± 1.2 years old	Before exercise protocol (study compared the outcomes before and after a 12-wk exercise regimen): - ↑ insulin - ↓ serum testosterone - ↓ sperm concentration - ↓ sperm progressive motility - ↓ sperm normal morphology	Walks and/or light jogging	↓ insulin (<than before EP) ↑ serum testosterone (>than before EP) ↑ sperm concentration (>than before EP) ↑ sperm progressive motility (>than before EP) ↑ sperm normal morphology (>than before EP)	[202]
**Ctrl group:** 16 wks sedentary (45 participants) **EP group:** 16 wks EP (45 participants)	Men with obesity; 25 to 40 years old	(no results—the study compares the effect of the EPs with sedentarism in men with obesity—Ctrl group is obese)	Treadmill exercise	↑ sperm concentration (>Ctrl) ↑ progressive motility (>Ctrl) ↑ sperm with normal morphology (>Ctrl) ↑ serum testosterone (>Ctrl)	[203]
**Ctrl group:** 14 wks no exercise (16 participants) **EP group:** 14 wks EP (+healthy diet) (27 participants)	Men with obesity; 20 to 59 years old	BBefore exercise protocol (study compared the outcomes before and after a 14-wk exercise regimen): - ↓ sperm count - ↓ sperm volume - ↑ Testosterone	Undisclosed	↑ Sperm count ↑ Semen volume ↑ Testosterone ↑ SHBG	[198]
**Rodent studies**					
**Ctrl group**: 12 wks SD & sedentary (*n =* 6) **EP group**: 12 wk SD & MIEP (*n =* 6) or HIEP (*n =* 6; continuous or interval HIEP) **HFD group**: 12 wks HFD & sedentary (*n =* 6) **HFD+EP groups**: 12 wks HFD & MIEP (*n =* 6) or HIEP (*n =* 6; continuous or interval HIEP)	Wistar rats	↓ GLUT-1 expression in SC ↓ GLUT-3 expression in SC ↓ MCT-4 expression in SC ↓ Igf1 expression in SC ↓ lactate and LDH levels in SC	Treadmill exercise (MIEP: 50–60% of Smax, HIEP: 70−75% of Smax, 60 min/day, 5 days/wk, 8 wks)	↑ GLUT-1 expression in SC (<Ctrl; MIEP only) (=Ctrl HIEP) ↑ GLUT-3 expression in SC (=Ctrl) ↑ MCT-4 expression in SC (<Ctrl) ↑ Igf1 expression in SC (<Ctrl) ↑ lactate and LDH levels in SC (<Ctrl; MIEP only) (=Ctrl HIEP)	[204]
**Ctrl group**: 21 wks SD (*n =* 15) **HFD group**: 21 wks HFD (*n =* 15) **HFD-EP groups**: 12 wks HFD + 1 wk AP + 8 wks MIEP (*n =* 15) or HIEP (*n =* 15)	C57BL/6 mice	↓ ST diameter, lumen diameter, epithelium height ↓ testis/body weight ratio ↓ sperm count ↓ sperm motility ↓ serum FSH ↓ serum testosterone ↓ serum LH ↑ serum estradiol ↓ SF-1, StAR, CYP11A1 and CYP17A1 ↑ lipid peroxidation ↑ antioxidant enzymes activity (SOD, CAT, GSH and GSH-Px) ↓ Nrf2, HO-1 and NQO expression ↑ testicular apoptosis ↑ m6A methylation	Treadmill exercise (MIEP: 50–60% of Smax, HIEP: 70−75% of Smax, 45 min/day, 5 days/wk, 8 wks)	↑ ST diameter, lumen diameter, epithelium height ↑ testis/body weight ratio (=Ctrl) ↑ sperm count (=Ctrl) ↑ sperm motility (=Ctrl) ↑ serum FSH (HIEP only) (=Ctrl) ↑ serum testosterone (HIEP only) (=Ctrl) No effect on LH or estradiol ↑ SF-1, StAR, CYP11A1 and CYP17A1 (=Ctrl) ↓ lipid peroxidation (HIEP> MIEP) (=Ctrl) ↑ Nrf2, HO-1 and NQO expression (HIEP>MIEP) (=Ctrl) ↓ testicular apoptosis ↓ m6A methylation	[142]
**Ctrl group:** 14 wks SD (*n =* 6) **HFD group:** 8 wks HFD (*n =* 6) **HFD-EP groups:** 8 wks HFD + 1 wk AP + 6 wks AEP (*n =* 6) or ANEP (*n =* 6)	Wistar rats	↓ serum FSH ↓ serum LH ↑ total oxidative status ↑ mitochondria-related genes (MFN2, PPARG, PGC1α, TXNL4B and PARP2)	Treadmill exercise (AP: 50–60% Smax, AEP: 50–90% Smax, 20 min/day,3 days/wk, 6 wks)	↑ serum FSH (=Ctrl) ↑ serum LH (=Ctrl) ↑ TAS (>Ctrl; AEP only) ↓ TOS (=Ctrl; AEP only) ↑ TOS (=HFD; ANEP only) ↓ mitochondria-related genes (MFN2, PPARG, PGC1α, TXNL4B and PARP2) (=Ctrl)	[205]
**Ctrl group**: 12 wk SD + 6 wks SD & sedentary (*n =* 7) **EP group**: 12 wk SD + 6 wks SD & EP (*n =* 7) **HFD group**: 12 wk HFD + 6 wks HFD & sedentary (*n =* 7) **HFD+EP group**: 12 wk HFD + 6 wks HFD & EP (*n =* 7)	Sprague Dawley rats	↓ serum LH ↓ serum FSH ↓ serum testosterone ↑ serum leptin (hyperleptinemia) ↑ testicular lipid peroxidation ↑ testicular DNA damage ↑ TNF-α and IL-6 levels ↑ myeloperoxidase activity ↑ atrophy of ST ↓ spermatogenesis (Johnsen’s score ↑ seminiferous tubules apoptosis	Swimming exercise (60 min/day, 5 days/wk, 6 wks)	↑ serum LH (=Ctrl) ↑ serum FSH (=Ctrl) ↑ serum testosterone (>Ctrl) ↓ serum leptin (=Ctrl) ↓ testicular lipid peroxidation (=Ctrl) ↓ testicular DNA damage (=Ctrl) ↓ TNF-α (>Ctrl) and IL-6 (<Ctrl) levels ↓ myeloperoxidase activity (>Ctrl) ↓ atrophy of ST ↑ spermatogenesis (Johnsen’s score) ↓ ST apoptosis	[206]
**Ctrl group:** 10 wks SD + 8 wks sedentary (*n =* 10) **HFD group:** 10 wks HFD + 8 wks sedentary (*n =* 10) **HFD+EP group:** 10 wks HFD + 8 wks MIEP (*n =* 12) or HIEP (*n =* 12)	C57BL/6L mice	↓ serum testosterone ↓ sperm count ↓ sperm motility ↑ sperm apoptosis ↓ TAS ↓ CAT, GSH-Px, GSH ↑ lipid peroxidation ↑ NO ↑ NF-κB, TNF-α, IL-1β, and IL-10 expression ↓ StAR, CYP11A1, SF-1 expression	Swimming exercise (MIEP: 20 min/day, once a day, HIEP: 20 min/day, twice a day, 5 days/wk, 8 wks)	↑ serum testosterone (=Ctrl; MIEP only) ↓ serum testosterone (=HFD; HIEP only) ↑ sperm count (=Ctrl; MIEP only) ↓ sperm count (=HFD; HIEP only) ↑ sperm motility (=Ctrl; MIEP only) ↓ sperm motility (=HFD; HIEP only) ↓ sperm apoptosis (=Ctrl; MIEP only) ↑ sperm apoptosis (=HFD; HIEP only) ↑ TAS (=Ctrl; MIEP only) ↓ TAS (=HFD; HIEP only) ↑ SOD (MIEP only) ↑ CAT, GSH-Px, GSH expression (=Ctrl; MIEP only) ↓ CAT, GSH-Px, GSH expression (=HFD; HIEP only) ↓ lipid peroxidation (=Ctrl; MIEP only) ↑ lipid peroxidation (=HFD; HIEP only) ↓ NO (=Ctrl; MIEP only) ↑ NO (=HFD; HIEP only) ↓ NF-κB, TNF-α, IL-1β, and IL-10 expression (=Ctrl; MIEP only) ↑ NF-κB, TNF-α, IL-1β, and IL-10 expression (=HFD; HIEP only) ↑ StAR, CYP11A1, SF-1 expression (=Ctrl; MIEP only) ↓ StAR, CYP11A1, SF-1 expression (=HFD; HIEP only)	[28]
**Ctrl group**: 12 wks SD & sedentary (*n =* 6) **EP group**: 12 wk SD & MIEP (*n =* 6) or HIEP (*n =* 6; continuous or interval HIEP) **HFD group**: 12 wks HFD & sedentary (*n =* 6) **HFD+EP groups**: 12 wks HFD & MIEP (*n =* 6) or HIEP (*n =* 6; continuous or interval HIEP)	Wistar rats	↓ SC number ↓ LC number ↑ Germ cells dissociation ↓ Spermatogenesis and spermiogenesis ↓ serum testosterone ↑ apoptosis in germ cells ↑ DNA fragmentation in germ cells	Treadmill exercise (MIEP: 50–60% Smax (0° incline), HIEP: 40–75% Smax with 20° incline, 80 min/day, 5 days/wk, 12 wks)	↑ SC number (<Ctrl) ↑ LC number (<Ctrl) ↓ Germ cells dissociation ↑ Spermatogenesis and spermiogenesis ↑ serum testosterone (<Ctrl) ↓ apoptosis in germ cells (>Ctrl; MIEP > HIEP) ↓ DNA fragmentation in germ cells (<Ctrl; MIEP > HIEP)	[207]
**Ctrl group**: 12 wk SD + 6 wks SD & sedentary (*n =* 8) **EP group**: 12 wk SD + 6 wks SD & MIEP (*n =* 8) **HFD group**: 12 wk HFD + 6 wks HFD & sedentary (*n =* 8) **HFD+EP group**: 12 wk HFD + 6 wks HFD & MIEP (*n =* 8)	Sprague Dawley rats	↓ total sperm count ↓ sperm progressive motility ↓ sperm with normal morphology ↑ sperm with midpiece defects ↑ sperm with tail defects ↑ epididymal epithelium degeneration * ↓ epididymal sperm accumulation	Swimming exercise (60 min/day, 5 days/wk, 6 wks)	↑ total sperm count (<Ctrl) ↑ sperm progressive motility (=Ctrl) ↑ sperm with normal morphology (=Ctrl) ↓ sperm with midpiece defects (=Ctrl) ↑ sperm with tail defects (=Ctrl) ↓ epididymal epithelium degeneration ↑ epididymal sperm accumulation	[208]
**Ctrl (SD+sedentary)**: 12 wks LFD + 9 wks sedentary **SD+EP**: 12 wks LFD + 1 wk AP + 8 wks EP **HFD+sedentary**: 12 wks HFD + 9 wks sedentary **HFD+EP**: 12 wks HFD + 1 wk AP + 8 wks EP	C57BL/6 mice	↓ sperm concentration ↓ sperm motility ↑ sperm protamine deficiency	Treadmill exercise (23 m/min, 45 min/day, 5 days/wk, 9 wks)	↑ sperm concentration (>Ctrl) ↑ sperm motility (>Ctrl) ↑ sperm lipid peroxidation (>Ctrl) ↓ sperm protamine deficiency (>Ctrl)	[209]
**Ctrl group:** 10 wks SD + 6 wks sedentary (*n =* 6) **HFD group:** 10 wks HFD + 6 wks sedentary (*n =* 6) **HFD+EP group:** 10 wks HFD + 6 wks MIEP (*n =* 6) or HIEP (*n =* 6)	C57BL/6J mice	↑ leptin (serum and testis) ↑ mRNA leptin ↓ leptin receptor signaling (↓ mRNA leptin receptor, JAK, STAT) ↑ serum estradiol ↓ serum testosterone ↓ testis/body mass ratio ↓ sperm count ↓ sperm motility ↓ SF1 ↓ steroidogenic enzymes (StAR, CYP11A1)	Swimming exercise (MIEP: 120 min/day, once a day, HIEP: 120 min/day, twice a day, 6 days/wk, 6 wks)	↓ leptin (serum and testis) (=Ctrl; HIEP > MIEP) ↓ mRNA leptin (=Ctrl) ↑ leptin receptor signaling (↓ mRNA leptin receptor, JAK, STAT) (=Ctrl) ↓ serum estradiol (=Ctrl) ↑ serum testosterone (MIEP only) ↑ testis/body mass ratio (=Ctrl) ↑ sperm count (MIEP only) ↑ sperm motility (MIEP only) ↑ SF1 (MIEP only) ↑ steroidogenic enzymes (StAR, CYP11A1) (MIEP only)	[210]
***Fa/Fa* group**: 10 wks sedentary (*n =* 7) ***Fa/Fa* + EP group**: 2 wks AP + 8 wks EP (*n =* 8) ***fa/fa* group**: 10 wks sedentary (*n =* 7) ***fa/fa* + EP group**: 2 wks AP +8 wks EP (*n =* 7)	Zucker rats	↑ insulin resistance ↓ serum total, serum-free and testicular testosterone ↑ serum and epidydimal adipose MCP-1	Treadmill exercise (20m/min, 60 min/day, 5 days/wk, 7 wks)	↓ insulin resistance compared to fa/fa (>Ctrl) ↑ serum total, serum-free and testicular testosterone (=Ctrl) ↓ serum and adipose MCP-1 (=Ctrl)	[211]
**Ctrl group:** 18 wks SD & sedentary (*n =* 8) **HFD group:** 18 wks HFD & sedentary (*n =* 8) **HFD+EP group:** 10 wks HFD + 8 wks EP (*n =* 8) **DC group:** 10 wks HFD + 8 wks SD & sedentary (*n =* 8) **DC+EP group:** 10 wks HFD + 8 wks SD % EP (*n =* 8)	C57BL6 mice	↓ sperm motility ↑ sperm with abnormal morphology (tail) ↑ sperm mitochondrial membrane potential	Swimming (60 min/day, 5 days/wk, 18 wks)	↑ sperm motility (=Ctrl) ↑ sperm abnormal tail morphology (=Ctrl) ↓ sperm mitochondrial membrane potential (>Ctrl)	[212]

Note: unless stated otherwise, the treadmill incline should be considered 0°. * Authors hypothesized it was due to oxidative stress, but did not assess parameters associated with oxidative stress. Abbreviations: ↑—increase; ↓—decrease; (=Ctrl)—no significant differences compared to the control group; (>Ctrl)—significantly increased compared to the control group; (<Ctrl)—significantly reduced compared to the control group; (=HFD)—no significant differences compared to the HFD group; 17β-HSD—17β-Hydroxysteroid dehydrogenases; 3β-HSD—3β-Hydroxysteroid dehydrogenase; ACP53—acetylation of lysine 379 in p53; AEP—aerobic exercise protocol; ANEP—anaerobic exercise protocol; AP—adaptative exercise protocol; Bax—BCL2 Associated X, Apoptosis Regulator; Bcl-2—B-cell lymphoma 2; CAT—catalase; COX-2—Cyclooxygenase-2; Ctrl—control; CYP—cytochrome P450; DC—diet change from standard diet to high fat diet; EP—exercise protocol; Fa/Fa—lean rats; fa/fa—rats with obesity induced by mutation; FGF-21—Fibroblast growth factor 21; FSH—follicle stimulating hormone; GLUT—Glucose transporter; GPx—glutathione peroxidase; GST—glutathione-S-transferase; HFD—high fat diet; HIEP—high-intensity exercise protocol; HIF-1α—hypoxia-inducible factor α-subunits; HO-1—heme oxygenase-1; Igf1—Insulin-like growth factor-1; IL—Interleukin; iNOS—nitric oxide synthases; JAK—Janus kinase; LC—Leydig cell; LDH—lactate dehydrogenase; LFD—low fat diet; LH—luteinizing hormone; LIEP—low-intensity exercise protocol; m6A—N6-methyladenosine; MCP-1—monocyte chemoattractant protein-1; MCT-4—Monocarboxylate transporter-4; MFN2—Mitofusin 2; MIEP—moderate-intensity exercise protocol; NF-κB—factor nuclear kappa B; NO—nitric oxide; NQO—NAD(P)H quinone oxidoreductase-1; Nrf2—nuclear factor erythroid 2–related factor 2; PARP2—Poly(ADP-Ribose) Polymerase 2; PGC1α—PPARG coactivator 1 alpha; PPARG—Peroxisome proliferator-activated receptor gamma; REP—resistance exercise protocol; ROS—reactive oxygen species; SC—Sertoli cells; SD—standard diet; SF-1—steroidogenic factor-1; SHBG—sex hormone binding globulin; SIRT1—sirtuin 1; Smax—maximum speed test; SOD—superoxide dismutase; ST—seminiferous tubules; StAR—steroidogenic acute regulatory protein; STAT—signal transducer and activator of transcription; TAS—total antioxidant status; TLR-4—Toll-like receptor 4; TNF-α—Tumor Necrosis Factor-alpha; TOS—total oxidant status; TXNL4B—Thioredoxin Like 4B.

**Table 3:** Summary of the main pathways addressed in this literature review.

Molecular Mechanism	Signaling Molecules (Up/Down)	References
Antioxidant response via Keap1-Nrf2-ARE and the Nrf2-SIRT1 axis	Nrf2 (↓ obesity), SIRT1 (↓ obesity), HO-1, NQO, CAT, SOD, GPx (↓ obesity; ↑ caloric restriction and physical exercise) progressive	[28,83,109,119,121,122,123,124,142,214,215]
Adipokine Imbalance:	Leptin (↑ obesity), Adiponectin (↓ obesity)	[46,47,49]
UPR	Adipose tissue UPR (↑ obesity)	[50,51,53,56]
Hypoxia	Hypoxia, HIF-1α, PAI-1 (↑ obesity, ↓ caloric restriction)	[53,57,169]
MCP-1 expression	MCP-1, macrophage infiltration in testis (↑ obesity, ↓ physical exercise)	[58,211]
NOX activity	NOX2, NOX4, NOX5 (↑ obesity)	[72,73,122]
Myeloperoxidase activity	Myeloperoxidase (↑ obesity, ↓ physical exercise)	[66,74,75,171,206]
Inflammatory cytokines and systemic inflammation	TNF-α, IL-6, IL-1β (↑ obesity, ↓ physical exercise), IL-10 (↓ obesity, ↑ physical exercise)	[28,32,83,186,190,192]
Steroidogenesis disruption		
Aromatase overexpression	Aromatase (↑ obesity), testosterone conversion to estrogens (↑ obesity)	[104,105,108]
TNF-α signaling	JNK/ERK/MAPK, NF-κB (↑ obesity), DAX-1 (↑ obesity), Nur77 and SF-1 (↓ obesity)	[[85,86,87,88]
Inhibition steroidogenic enzymes	StAR, CYP11a, CYP17a, 3β-HSD (↓ obesity)	[104,105,106,107,108,142,210]
Transgenerational Effects	Altered metabolic, lipidic, and sncRNA testicular fingerprint	[150,151,152,153,154,178,221,222]

Abbreviations: ↑—increase; ↓—decrease; 3β-HSD—3β-Hydroxysteroid dehydrogenase; ARE—antioxidant response element; CAT—catalase; CYP—cytochrome P450; GPx—glutathione peroxidase; HIF-1α—hypoxia-inducible factor 1α; HO-1—heme oxygenase-1; IL—Interleukin; JNK/ERK/MAPK—Jun N-terminal kinase 1/extracellular regulated protein kinases/mitogen-activated protein kinase; Keap1—Kelch-like ECH-associated protein 1; MCP-1—monocyte Chemoattractant Protein-1; NF-κB—factor nuclear kappa B; NOX—nicotinamide adenine dinucleotide phosphate (NADPH) oxidase; NQO—NADPH:quinone oxidoreductase; Nrf2—nuclear factor erythroid 2–related factor 2; Nur77—nuclear receptor subfamily 4 group A member 1; PAI-1—plasminogen activator inhibitor; SF-1—steroidogenic factor 1; SIRT1—sirtuin 1; sncRNA—small noncoding RNAs; SOD—superoxide dismutase; TNF-α—Tumor Necrosis Factor-alpha; UPR—unfolded protein response.

## Data Availability

All of the data are contained within this article and can be discussed by the authors without undue reservation.
